# Global Transcriptional Profiling Reveals Novel Autocrine Functions of Interleukin 6 in Human Vascular Endothelial Cells

**DOI:** 10.1155/2020/4623107

**Published:** 2020-04-28

**Authors:** Liza U. Ljungberg, Mulugeta M. Zegeye, Caroline Kardeby, Knut Fälker, Dirk Repsilber, Allan Sirsjö

**Affiliations:** School of Medical Sciences, Cardiovascular Research Centre, Örebro University, Örebro, Sweden

## Abstract

**Background:**

Interleukin 6 (IL6) is a multifunctional cytokine produced by various cells, including vascular endothelial cells. IL6 has both pro- and non-/anti-inflammatory functions, and the response to IL6 is dependent on whether it acts via the membrane-bound IL6 receptor *α* (IL6R*α*) (classic signaling) or the soluble form of the receptor (transsignaling). As human endothelial cells produce IL6 and at the same time express IL6R*α*, we hypothesized that IL6 may have autocrine functions.

**Methods:**

Knockdown of IL6 in cultured human endothelial cells was performed using siRNA. Knockdown efficiency was evaluated using ELISA. RNA sequencing was employed to characterize the transcriptional consequence of IL6 knockdown, and Ingenuity Pathway Analysis was used to further explore the functional roles of IL6.

**Results:**

Knockdown of IL6 in cultured endothelial cells resulted in a 84-92% reduction in the release of IL6. Knockdown of IL6 resulted in dramatic changes in transcriptional pattern; knockdown of IL6 in the absence of soluble IL6R*α* (sIL6R*α*) led to differential regulation of 1915 genes, and knockdown of IL6 in the presence of sIL6R*α* led to differential regulation of 1967 genes (fold change 1.5, false discovery rate < 0.05). Pathway analysis revealed that the autocrine functions of IL6 in human endothelial cells are mainly related to basal cellular functions such as regulation of cell cycle, signaling, and cellular movement. Furthermore, we found that knockdown of IL6 activates functions related to adhesion, binding, and interaction of endothelial cells, which seem to be mediated mainly via STAT3.

**Conclusion:**

In this study, a large number of novel genes that are under autocrine regulation by IL6 in human endothelial cells were identified. Overall, our data indicate that IL6 acts in an autocrine manner to regulate basal cellular functions, such as cell cycle regulation, signaling, and cellular movement, and suggests that the autocrine functions of IL6 in human endothelial cells are mediated via IL6 classic signaling.

## 1. Background

The vascular endothelium is crucial to maintain normal vascular homeostasis. A healthy endothelium has vasodilating, anti-inflammatory, and antithrombotic properties. However, during vascular diseases (e.g., atherosclerosis), the endothelial function is impaired, which is usually referred to as endothelial dysfunction and is associated with proinflammatory and prothrombotic properties, as well as reduced vasodilatory capacity [1]. Endothelial cells express a large number of genes and regulatory proteins both in healthy conditions and during vascular disease, including pro- and anti-inflammatory cytokines [2]. Interleukin 6 (IL6) is a multifunctional cytokine produced by various cells, including vascular endothelial cells [3–5]. IL6 plays a central role in inflammation by controlling differentiation, proliferation, migration, and apoptosis of targeted cells, and it has been implicated in the pathology of a number of diseases, such as rheumatoid arthritis, systemic lupus erythematosus, and atherosclerosis [6, 7]. IL6 binds to its specific and nonsignaling receptor IL6 receptor *α* (IL6R*α*), which forms a dimeric complex with the signaling-transducing receptor gp130 (IL6ST) which in turn mediates a signal [8]. While gp130 is ubiquitously expressed, the expression of IL6R*α* is restricted to certain cell types (e.g., hepatocytes and leukocytes) [9]. However, IL6R*α* also exists in a soluble form (sIL6R*α*) which is generated from alternative splicing or enzymatic cleavage from the plasma membrane [10, 11]. The sIL6R*α* forms a complex with IL6, and this complex binds to gp130/IL6ST which then mediates a signal, also in cells that do not express IL6R*α* [12]. Thus, cells that lack IL6R*α* can respond to IL6 if the soluble form of IL6R*α* is present. Signaling via the soluble form of IL6R*α*, usually referred to as IL6 transsignaling, mainly mediates proinflammatory responses, while signaling via the membrane-bound form (referred to as IL6 classic signaling) is considered to mediate non- or anti-inflammatory responses [12].

IL6 mainly acts in an autocrine and intracrine fashion in many leukocytes that express both IL6 and membrane bound IL6R*α* [13, 14]. Similarly, an autocrine function of IL6 has been shown in endothelial cells, but only in the presence of an exogenous sIL6R*α*. However, we have recently demonstrated that vascular endothelial cells also express functional IL6R*α* capable of mediating intracellular signaling [5], suggesting a true autocrine effect mediated by the membrane-bound IL6R*α* receptor on the endothelial surface.

In this study, we evaluated the autocrine roles of IL6 in human vascular endothelial cells using siRNA-mediated IL6 gene silencing in cultured endothelial cells. RNA sequencing was employed to characterize the transcriptional activities of IL6 in vascular endothelial cells, and Ingenuity Pathway Analysis (IPA) was used to further explore the functional roles of IL6.

## 2. Methods

In this study, we performed knockdown of IL6 in human endothelial cells followed by RNA sequencing, in order to unravel the autocrine functions of IL6.

### 2.1. Cell Culture

Human umbilical vein endothelial cells (HUVECs, pooled from several donors) (Gibco, Thermo Fisher Scientific, Waltham, MA, USA) were cultured in VascuLife basal medium supplemented with Vascular Endothelial Growth Factor (VEGF) LifeFactors Kit (Lifeline Cell Technology, Frederick, MD, USA), penicillin 0.1 U/ml (Gibco), and streptomycin 100 ng/ml (Gibco). The cells were grown in 75 cm^2^ cell culture flasks (Sarstedt, Nümbrecht, Germany) and kept at 37°C in 95% air and 5% CO_2_. Medium was replaced every 48-72 h, and the cells were subcultured when confluent. Cells in passages 5-7 were used.

### 2.2. siRNA-Mediated Gene Silencing of IL6 in HUVECs

Knockdown of IL6 in HUVECs was performed using siRNA and Lipofectamine. HUVECs were seeded at a density of 200,000 cells per well in 6-well plates (Sarstedt) in VascuLife basal medium supplemented with VEGF LifeFactors Kit, penicillin 0.1 U/ml, and streptomycin 100 ng/ml and incubated at 37°C in 95% air and 5% CO_2_ overnight. The following morning, the cells were rinsed once in Opti-MEM I reduced serum medium (Gibco), followed by the addition of 700 *μ*l Opti-MEM I reduced serum medium containing 4 *μ*l Lipofectamine 2000 (Thermo Fisher Scientific, Waltham, MA, USA) and 20 pmol of 3 IL6-targeting Stealth RNAi™ siRNAs (Thermo Fisher Scientific) or 60 pmol nontarget Stealth RNAi™ siRNA negative control (Thermo Fisher Scientific) per well. The plates were incubated at 37°C in 95% air and 5% CO_2_ for 4 hours followed by the addition of 1.3 ml VascuLife basal medium supplemented with VEGF LifeFactors Kit per well. Knockdown of IL6 was performed both in the presence and absence of the soluble IL6 receptor, in four replicates, accompanied by control samples without knockdown. Recombinant human sIL6R*α* 100 ng/ml (R&D Systems, Minneapolis, MN, USA) was added to the wells 6 h after introducing siRNA. Cells and cell culture medium were collected after 48 h and stored at -80°C pending analysis. Cell culture medium was used for the analysis of IL6 released into the medium, while cells were used for isolation of RNA.

### 2.3. Blocking of IL6-Transsignaling Using sgp130Fc Fusion Protein

HUVECs were treated with sgp130Fc fusion protein 1 *μ*g/ml (R&D Systems) which specifically inhibits IL6 transsignaling, alone or in combination with (1) recombinant IL6 (50 ng/ml) or (2) recombinant IL6 (50 ng/ml) together with recombinant sIL6R*α* (10 ng/ml). HUVECs were exposed for 30 min for the determination of the phosphorylation of STAT3, AKT, or ERK1/2 using Western Blot, and for 48 h for gene expression analysis.

### 2.4. Enzyme-Linked Immunosorbent Assay (ELISA)

The release of IL6 into the cell culture medium was analyzed using DuoSet® ELISA (R&D Systems) according to the manufacturer's instructions. Absorbance measurements were performed at 450 nm, with a wavelength correction at 540 nm using a Multiscan Ascent Spectrophotometer (Lab Systems, Thermo Fisher Scientific).

### 2.5. RNA Sample Preparation and Sequencing

RNA was isolated from HUVECs using E.Z.N.A.® Total RNA Kit I (Omega Bio-tek, Norcross, GA, USA) according to the manufacturer's instructions. The cell lysate was treated with PureLink® DNAse (Thermo Fisher Scientific) for 15 min after being loaded onto the column. RNA was eluted in RNAse-free water, and RNA concentration was measured using NanoDrop 2000 (Thermo Fisher Scientific).

#### 2.5.1. Library Preparation

Sequencing libraries were prepared from 1 *μ*g total RNA using the TruSeq Stranded mRNA Sample Prep Kit including poly-A selection (Illumina, San Diego, CA, USA) according to the manufacturer's protocol (#15031047 revE). Briefly, the poly-A-selected RNA was fragmented and the RNA was reverse transcribed into cDNA using random-sequence primers and SuperScript™ III Reverse Transcriptase (Thermo Fisher Scientific). The cDNA was purified using AMPure XP beads (Beckman Coulter, Brea, CA, USA) and amplified for 15 cycles of PCR with barcoded primers, followed by purification using AMPure beads. The quality of the libraries was evaluated using the Fragment Analyzer (Advanced Analytical Technologies, Ankeny, IA) with the dsDNA Reagent Kit (Advanced Analytical Technologies). The adapter-ligated fragments were quantified by qPCR using the Library Quantification Kit for Illumina (KAPA Biosystems, Wilmington, MA) on a StepOnePlus instrument (Applied Biosystems, Thermo Fisher Scientific) prior to cluster generation and sequencing.

#### 2.5.2. Cluster Generation and Sequencing

Sequencing was performed by the SNP&SEQ Technology Platform, a national facility within the National Genomics Infrastructure (NGI), hosted by Science for Life Laboratory, in Uppsala, Sweden (https://www.scilifelab.se/facilities/ngiuppsala/). Sequencing was carried out on an Illumina HiSeq2500 instrument (HCS version 2.2.58/RTA version 1.18.64) according to the manufacturer's instructions, in which they were sequenced with 50 bp paired-end reads (60-80 million reads per sample). Demultiplexing and conversion to FASTQ format was performed using the CASAVA software (version 1.8.4) provided by Illumina. Additional statistics on sequencing quality were compiled with an in-house script from the FASTQ-files, RTA, and CASAVA output files.

### 2.6. cDNA Synthesis

cDNA was synthesized from 1 *μ*g RNA in 20 *μ*l reactions using a high-capacity cDNA reverse transcription kit (Applied Biosystems, Thermo Fisher Scientific) according to the manufacturer's instructions. cDNA was synthesized using the following thermal cycles: 10 min at 25°C, 120 min at 37°C, and 5 min at 85°C. Samples were cooled down to 4°C and stored at -20 until analysis.

### 2.7. Quantitative Real-Time PCR

Gene expression analysis of selected genes was performed using quantitative real-time PCR (qPCR). A six-point standard curve was prepared by making 1 : 2 dilutions of pooled cDNA. qPCR was performed in 10 *μ*l reactions containing LuminoCt® qPCR ReadyMix™ (Sigma-Aldrich, St. Louise, MO, USA), Taqman primers/probes (Applied Biosystems, Thermo Fisher Scientific), cDNA, and water. The following cycling conditions were used: initialization at 95°C for 20 s, followed by 40 cycles at 95°C for 1 s and 60°C for 20 s in the QuantStudio 7 Flex Real-Time PCR System (Applied Biosystems, Thermo Fisher Scientific). Relative quantity was calculated based on the standard curve and normalized to the expression of the housekeeping gene (GAPDH).

### 2.8. Western Blotting

Cells were lysed using ice-cold RIPA buffer (Millipore, Burlington, MA, USA). Protein amount was measured using Micro BCA™ Protein Assay (Pierce, Thermo Fisher Scientific) according to the manufacturer's instructions. Absorbance was measured at 562 nm using Cytation 3 Imaging Reader (BioTek Instruments Inc., Winooski, VT, USA) to obtain protein concentration. Cell lysates were mixed with SDS sample buffer, and proteins were denatured at 95°C for 5 min. Proteins (10-15 *μ*g) were separated on 4-12% NuPAGE® Novex Bis-Tris Gels (Invitrogen, USA) using MOPS running buffer (Invitrogen, USA). A protein ladder (MagicMark™ XP Western Protein Standard, Invitrogen) was included to determine the molecular masses of the proteins. Proteins were blotted onto Immun-Blot™ PVDF membranes (0.2 *μ*m) (Bio-Rad, Hercules, CA, USA), and membranes were blocked in 5% BSA in TBS-T (10 mM Tris-HCl, pH 8.0; 150 mM NaCl; and 0.1% (*w*/*v*) Tween-20). Subsequent washing steps were performed using TBS-T. For assessing STAT3 phosphorylation, membranes were probed with anti-phospho-Stat3Tyr705 antibody (Cell Signaling Technology, Danvers, MA, USA, #9131; 1 : 1000 dilution) diluted in 5% BSA in TBS-T. Membranes were also probed for *β*-tubulin using an anti-*β*-tubulin antibody (Millipore, #05-661; 1 : 2000 dilution) diluted in 5% BSA in TBS-T, which serve as a loading control. The membranes were then incubated with horseradish peroxidase- (HRP-) conjugated goat anti-rabbit IgGs (Cell Signaling Technology, #7074; 1 : 2000) or horse anti-mouse IgGs (Cell Signaling Technology, #7076; 1 : 2000). Membranes were subsequently covered with Immobilon™ Western Chemiluminescent HRP Substrate solution (Millipore, USA), and chemiluminescence was detected using LI-COR Odyssey Fc Imaging System (LI-COR Biotechnology UK Ltd., United Kingdom). Quantification of protein bands was performed using Image Studio Software (LI-COR Biotechnology).

### 2.9. Data Analysis of Gene Expression Sequencing Data

All 16 samples (with/without soluble receptor, with/without knockdown, 4 replicates) passed fastQC quality control [15]. Sequences were processed with cutadapt [16] to remove adapter sequences and to filter bad quality sequences. Sequences of a minimum length of 20 nt and with at least a quality score of 20 were kept. Trimmed and filtered reads were then mapped on the human transcriptome using the STAR aligner [17] together with the ENSEMBL human genome/transcriptome GRCh38 sequence and annotation files available from the ENSEMBL website [18] (ftp://ftp.ncbi.nlm.nih.gov/genomes/all/GCA/000/001/405/GCA_000001405.15_GRCh38/seqs_for_alignment_pipelines.ucsc_ids/GCA_000001405.15_GRCh38_no_alt_analysis_set.fna.gz). Samtools was used to convert aligned sequences to BAM format [19].

### 2.10. Analysis of Differential Gene Expression

Raw count data for all samples were joined into a common data table and then further analyzed using the edgeR package. First, read numbers were normalized with respect to total numbers of reads and compositional differences between the libraries [20], followed by fitting a two-factorial block design (knockdown/no knockdown, and experimental block in 1 : 4, representing the four replicates) with a linear model, separately for the cases of the soluble receptor being present or not [21].

### 2.11. Statistical Analysis of ELISA Data

One-way ANOVA followed by Bonferroni's post hoc test was used to analyze release of IL6 determined by ELISA. Graphic illustrations and statistical analyses were generated from GraphPad Prism version 5.01 (GraphPad Software, La Jolla, CA, USA).

### 2.12. Ingenuity Pathway Analysis

Ingenuity Pathway Analysis (IPA, QIAGEN Bioinformatics, April 4th 2017) was used to analyze the impact of IL6 knockdown on pathways and gene group level [22]. Three different comparisons were made: (1) impact of IL6 knockdown in the absence of the soluble IL6 receptor, (2) impact of IL6 knockdown in the presence of the soluble IL6 receptor, and (3) impact of the soluble IL6 receptor alone. For each comparison, a core analysis was performed. Genes were filtered for fold change ± 1.5 and Benjamini-Hochberg's false discovery rate (FDR) < 0.05. From the core analysis, canonical pathways as well as diseases and functions with a *z*-score of >2 or <-2 were considered significant. The number of regulated genes within each pathway was determined and used to calculate the ratio of regulated genes. All enriched canonical pathways with their corresponding differentially regulated genes were plotted in order to evaluate the overlap between the pathways.

All significantly regulated diseases and biofunctions were classified based on what main category they belong to in the Ingenuity Knowledge Base [22] and the number of activated and inhibited functions in each category were plotted. Since some categories overlap, some functions or diseases were classified into more than one category. Only categories containing 3 or more functions were included.

Furthermore, all differentially regulated genes from the enriched functions in the category “Cardiovascular System Develop and Function” were used to generate a merged network. There was an almost complete overlap between the 5 enriched functions, from which “Interaction of endothelial cells” included all the 42 differentially regulated genes found in the other 4 functions. These 42 genes, together with the known “core genes” crucial for IL6 signaling (IL6, IL6R, IL6ST, STAT3, JAK1, JAK2, and SOCS3) were included in the network. The merged network was generated by connecting all 42 genes using only direct connections, which have been experimentally observed in human samples. The IL6 “core genes” were connected with the same settings resulting in two independent networks. Subsequently, these two networks were merged using “Path Explorer,” allowing direct connections, experimentally observed in human samples, and allowing for a maximum of one node of individual molecules, excluding chemical and biological drugs. Four molecules remained unconnected (TSTA3, FOLH1, mir-126, and S1PR2). Those molecules were removed from the network.

In order to evaluate whether the genes found to be differentially regulated after IL6 knockdown in human endothelial cells were previously known to be regulated by IL6, we used IPA and the STRING database [23] to identify known interaction partners for IL6. In the STRING database (version 10), we selected direct and indirect interaction partners (first and second neighbors), using experiments and databases as sources, with a confidence of 0.9 in human samples. In IPA, we evaluated direct and indirect interaction partners including upstream and downstream regulators, allowing for one node, and evidence from experiments in human samples (17th October 2019).

## 3. Results

**Figure 1:**
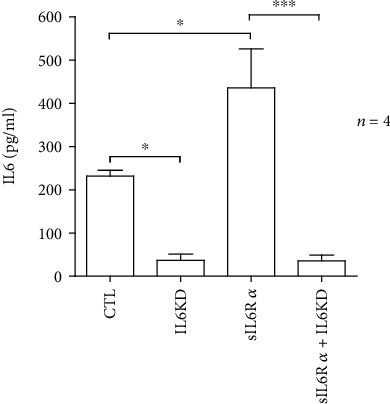
siRNA-mediated knockdown of interleukin 6 (IL6) in human vascular endothelial cells (HUVECs). Knockdown was performed in the presence and absence of the soluble IL6 receptor (sIL6R*α*) (100 ng/ml). The release of IL6 into the cell culture medium was measured using ELISA 48 h after siRNA was introduced. Data from four independent experiments performed in duplicate are shown. ^∗^*p* < 0.05 and ^∗∗∗^*p* < 0.001.

Knockdown of IL6 in human vascular endothelial cells was performed using siRNA. Knockdown was confirmed in the cell culture medium by ELISA, 48 h after introducing siRNA targeting IL6 (Figure 1). Knockdown efficiency was 84% and 92% in the absence and presence of sIL6R*α*, respectively. Treatment with sIL6R*α* alone resulted in 88% increased release of IL6 into the medium.

### 3.1. Transcriptional Regulation of Genes by IL6 Knockdown in Human Vascular Endothelial Cells

RNA sequencing was performed on human vascular endothelial cells after IL6 knockdown, both in the presence and absence of sIL6R, in order to evaluate the autocrine functions of IL6 in human endothelial cells. Differentially expressed genes were defined as a fold change of >1.5 and a FDR < 0.05. Knockdown of IL6 in the absence of sIL6R*α* resulted in 1915 differentially expressed genes (886 upregulated, 1029 downregulated), and knockdown of IL6 in the presence of sIL6R*α* resulted in 1967 differentially expressed genes (903 upregulated, 1064 downregulated). The top 25 up- and downregulated genes after IL6 knockdown in the absence and presence of sIL6R*α* are shown in Supplementary Tables 1–2. Among the most upregulated genes after IL6 knockdown, we find cytokines and cytokine receptors (IL1A, TGFB2, and TGFBR1), proteins important for cell adhesion (NEGR1, PDZD2, and LGALS3BP), and proteins that regulate cell differentiation (DACT1, MEDAG), while the most downregulated genes were genes involved in cell cycle regulation (ESCO2, MCM10, and SKP2), transcriptional regulators (E2F2, PRRX1), and different transporters (RAB27B, SLC7A14, and SLC44A4). Treatment with sIL6R*α* alone had limited effect and resulted in only 59 differentially expressed genes (51 upregulated, 8 downregulated) (Supplementary Table 3).

**Figure 2:**
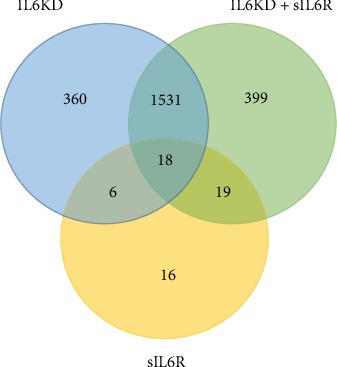
Number of differentially expressed genes in human endothelial cells after IL6 knockdown. The number in each circle represents the number of differentially expressed genes using a fold change of 1.5 and Benjamini-Hochberg's estimate of the false discovery rate below 0.05. The numbers within intersections stand for mutual differentially expressed genes between the different conditions, and numbers outside intersections represent genes unique to each condition. IL6KD: IL6 knockdown vs. control (blue circle). IL6KD+sIL6R: IL6 knockdown in the presence of the soluble IL6 receptor vs. soluble IL6 receptor (green circle). sIL6R: soluble IL6 receptor vs. control (yellow circle).

As shown in the Venn diagram (Figure 2), there is a major overlap between genes differentially regulated by IL6 knockdown in the absence and presence of sIL6R*α*. In total, 1549 genes were common in the two conditions, corresponding to approximately 80% of all differentially expressed genes.

**Figure 3:**
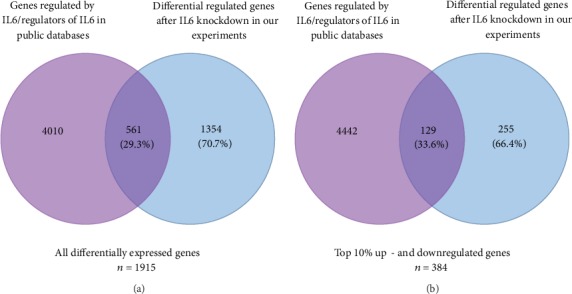
Knockdown of IL6 in human endothelial cells lead to identification of a large number of novel IL6-regulated genes. Comparison of lists of genes differentially regulated after IL6 knockdown in human endothelial cells with lists of known interaction partners of IL6 found in public databases (the Ingenuity Knowledge Base® (IPA) and the STRING database). Numbers in intercepts stand for known interaction partners of IL6 found in either IPA or in STRING and differentially regulated by IL6 knockdown in human endothelial cells in our experiments. Numbers outside intersections represent known interaction partners for IL6 found in IPA or STRING but not differentially regulated after IL6 knockdown (in purple) and differentially regulated genes after IL6 knockdown not described as interaction partners in IPA or STRING (in blue). (a) All differentially regulated genes after IL6 knockdown (*n* = 1915). (b) The top 10% most up- or downregulated genes after IL6 knockdown.

In order to evaluate whether the genes differentially regulated after IL6 knockdown in human endothelial cells were previously known to be regulated by IL6, we used IPA and the STRING database to identify known regulators and regulatory targets for IL6. Of the 1915 differentially regulated genes after IL6 knockdown found in our data, 1354 (70.7%) were not previously described as IL6 neighbors in the STRING database or Ingenuity Knowledge Base (Figure 3(a)). A full list of genes can be found in Supplementary Table 7. In addition, for the 10% most up- and downregulated genes, a slightly lower proportion of the genes (66.4%, *n* = 255) were previously not identified as first or second neighbors in the STRING or Ingenuity Knowledge Base (Figure 3(b)).

### 3.2. Transcriptional Regulation of IL6 Core Genes

**Figure 4:**
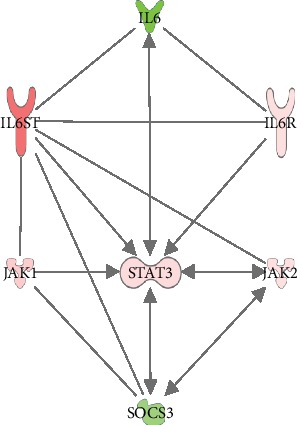
Knockdown of IL6 in human endothelial cells affects the gene expression of proteins known to be involved in IL6-mediated signaling. Knockdown of IL6 was performed using siRNA-mediated gene silencing followed by RNA sequencing in cultured human vascular endothelial cells. The impact of IL6 knockdown on genes known to be central in IL6 signaling (IL6 core genes) was investigated. Green symbols indicate downregulated genes, and red/pink symbols indicate upregulated genes. Pathway and coloring were generated using Ingenuity Pathway Analysis software.

As expected, knockdown of IL6 affects the transcription of genes known to be involved in IL6 signaling (referred to as “IL6 core genes”). As shown in Figure 4 (and Supplementary Table 4), IL6R and IL6ST/gp130 as well as STAT3, JAK1, and JAK2 were upregulated after IL6 knockdown. On the contrary, SOCS3, which is a negative regulator of IL6 signaling, was downregulated by IL6 knockdown. A very similar pattern was seen for IL6 knockdown in the presence of the IL6 receptor (data not shown).

### 3.3. IL6 Classic Signaling and Transsignaling

**Figure 5:**
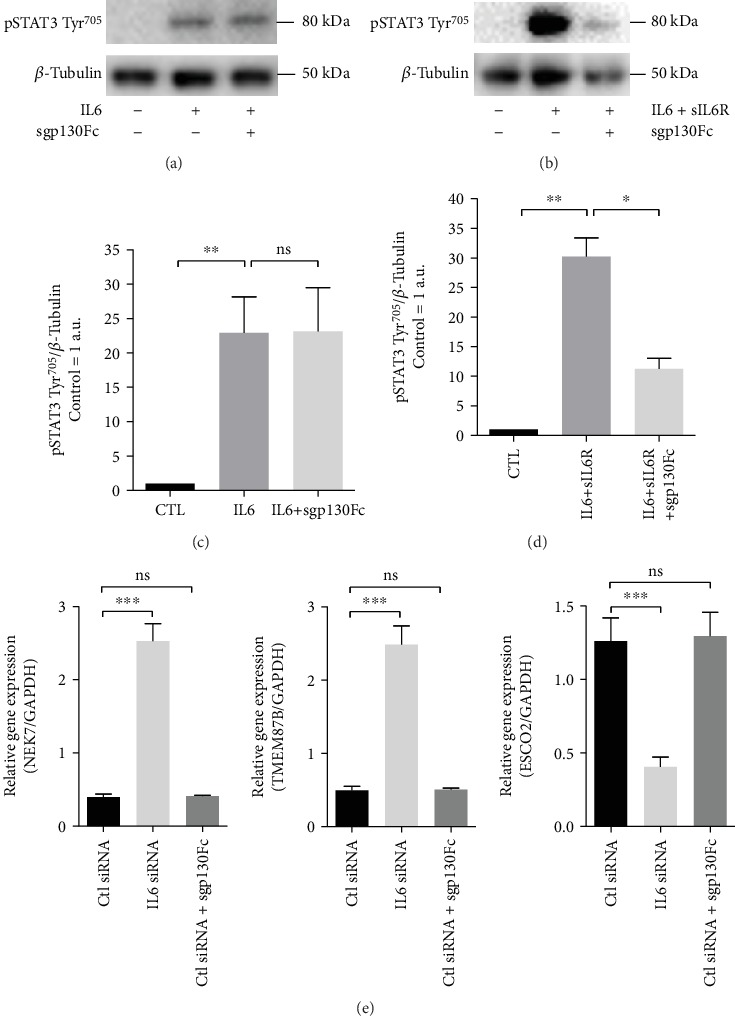
Specific blockage of IL6-transsignaling does not affect genes differentially regulated after IL6 knockdown in human endothelial cells. Soluble gp130 Fc fusion protein (sgp130Fc) was used to evaluate whether specific blockage of IL6 transsignaling affects the gene expression of 3 selected genes differentially regulated after IL6 knockdown. sgp130Fc does not affect the phosphorylation of STAT3 induced by IL6 classic signaling (a, c) but inhibits phosphorylation of STAT3 induced by IL6 transsignaling (IL6+sIL6R) (b, d). Gene expression of three genes differentially regulated after IL6 knockdown (NEK7, TEMEM87B, and ESCO2) was not affected by sgp130Fc (e). Data from 3 to 4 independent experiments are shown. Phosphorylation of STAT3 was evaluated after 30 min treatments and gene expression after 48 h. IL6 was used at a concentration of 50 ng/ml, sIL6R at 10 ng/ml, and sgp130Fc at 1 *μ*g/ml. ^∗^*p* < 0.05, ^∗∗^*p* < 0.01, and ^∗∗∗^*p* < 0.001.

Next, we evaluated whether the differentially expressed genes after IL6 knockdown are regulated via IL6 classic signaling or transsignaling. HUVECs were treated with the sgp130Fc fusion protein (sgp130Fc), which specifically blocks IL6 transsignaling [24]. First, we verified that sgp130Fc actually does block IL6-mediated transsignaling in our model. As shown in Figure 5, sgp130Fc significantly reduces IL6 transsignaling- (IL6+sIL6R-) mediated STAT3 phosphorylation, while IL6 classic signaling- (IL6 alone) induced STAT3 phosphorylation is not affected. Then, gene expression of three selected genes from the most differentially expressed genes (shown in Supplementary Table 1) were analyzed in sgp130Fc-treated samples as well as in IL6 knockdown samples and untreated controls. As shown in Figure 5, none of the genes were affected by sgp130Fc, indicating that these genes are regulated by IL6 classic signaling.

### 3.4. Functional Analysis of Genes Regulated by IL6 Knockdown

**Figure 6:**
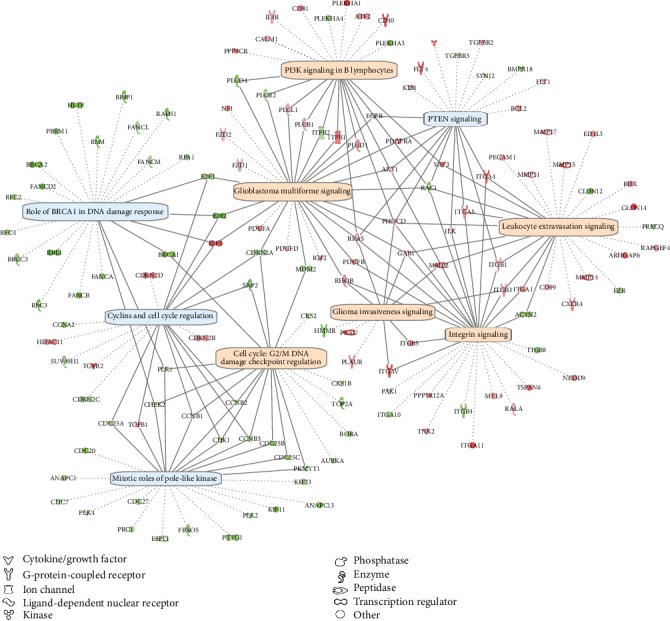
Canonical pathways regulated by IL6 knockdown. Using Ingenuity Pathway Analysis (IPA), enriched canonical pathways after IL6 knockdown in human endothelial cells were plotted together with the differentially expressed genes within each pathway. Activated pathways (*z* − score > 2) are indicated in orange, and inhibited pathways (*z* − score<−2) are indicated in blue. Downregulated genes are indicated in green, while upregulated genes are indicate in red. Genes only included in one pathway are indicated by dashed lines, and genes included in more than one pathway are indicated by solid lines. Only significantly regulated pathways (*p* < 0.05) are included, and only differentially regulated genes (fold change > 1.5, Benjamini-Hochberg's estimated false discovery rate < 0.05) in those pathways are shown.

In order to elucidate what functions are regulated by IL6 in vascular endothelial cells, we performed gene ontology [25] enrichment analysis using Ingenuity Pathway Analysis. A total of ten canonical pathways were found enriched by differentially expressed genes after IL6 knockdown (Supplementary Table 5). After applying Benjamini-Hochberg's FDR threshold at 5%, six of those GO gene sets remained significantly enriched. Knockdown of IL6 in the presence and absence of sIL6R*α* showed a similar pattern (data not shown), where most of the pathways were enriched in both conditions. In order to investigate to what extent the enriched pathways overlap (i.e., if the same group of genes are included in several of the enriched pathways), the ten enriched pathways and their differentially expressed genes after IL6 knockdown in the absence of sIL6R*α* were plotted (Figure 6). A total of 140 differentially expressed genes were included in the ten enriched pathways, and 95 of those genes (68%) were exclusively found in one pathway. The remaining 45 genes (32%) were present in two or more pathways, while 19 genes (14%) were belonging to three or more pathways. The corresponding numbers for the six enriched pathways that remain after applying Benjamini-Hochberg's FDR threshold at 5% are as follows: there were 89 genes in total, 67 of those were unique (75%), 22 genes (25%) were found in two or more pathways, and 11 genes (12%) belonged to three or more pathways.

**Figure 7:**
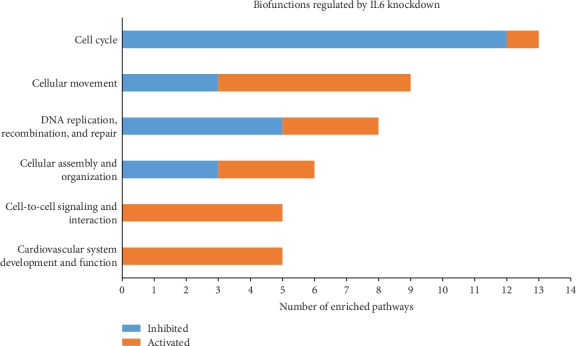
Functions regulated by IL6 knockdown in human endothelial cells. Using Ingenuity Pathway Analysis (IPA), diseases and biofunctions regulated by IL6 knockdown in human endothelial cells were evaluated. Diseases and biofunctions were classified into different categories based on the Ingenuity Knowledge Base classification. Activated diseases or biofunctions are indicated in orange, and inhibited diseases or biofunctions are indicated in blue. Categories which include 3 or more functions are shown. Functions with Benjamini-Hochberg's estimated false discovery rate < 0.05 and *z* − score<−2 or >2 were considered as enriched.

Next, we evaluated diseases and biofunctions that were enriched after IL6 knockdown. In total, 32 functions were found enriched after IL6 knockdown in the absence of sIL6R*α* and 34 in the presence of sIL6R*α* (Supplementary Table 6). Similar to what was seen for the canonical pathways, knockdown of IL6 in the presence and absence of sIL6R*α* showed a similar pattern, where most of the functions were enriched in both conditions. All significantly enriched functions or diseases were classified based on what main category they belong to in the Ingenuity Knowledge Base, and the number of activated and inhibited functions in each category were plotted. As shown in Figure 7, the enriched biofunctions included cell cycle; cellular movement; DNA replication, recombination, and repair; cellular assembly and organization; cell-to-cell signaling and interaction; and cardiovascular system development and function.

**Figure 8:**
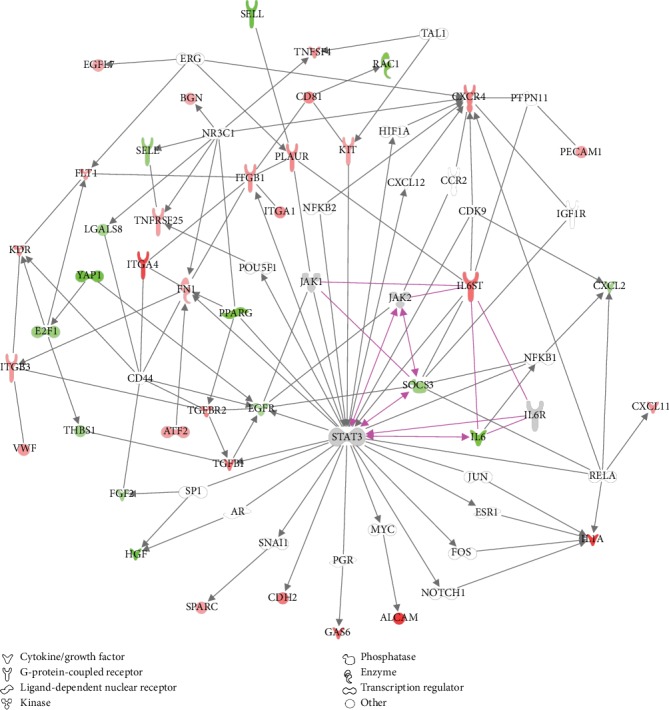
Merged network showing the interplay between IL6-mediated signaling and interaction, binding, and adhesion of endothelial cells. Using Ingenuity Pathway Analysis (IPA), a merged network was generated by connecting genes known to be central for IL6-mediated signaling (IL6, IL6R, IL6ST, STAT3, JAK1, JAK2, and SOCS3) with 42 differentially regulated genes extracted from the enriched functions related to binding, adhesion, and interaction of endothelial cells which were predicted to be activated by IL6 knockdown. Downregulated genes are indicated in green while upregulated genes are indicated in red using a fold change of 1.5 and Benjamini-Hochberg's estimated false discovery rate < 0.05. Grey symbols indicate expressed genes which were not differentially regulated after IL6 knockdown, while white symbols indicate nonexpressed genes. Purple lines indicate the interaction between genes known to be central for IL6 signaling.

We further investigated the functions belonging to the category “Cardiovascular System Development and Function.” As shown in Table 1, all the five functions in this category are activated by IL6 knockdown and they are all related to endothelial interaction, binding, or adhesion of endothelial cells. There is a considerable overlap of all these five functions, and the 42 differentially expressed molecules in the function “interaction of endothelial cells” cover all the differentially expressed genes included in the other four functions. A network was generated using the 42 differentially expressed genes from the “interaction of endothelial cells” function together with the IL6 core genes (Figure 8).

This network shows that most of the interactions between the IL6 core genes and the genes involved in endothelial interaction, adhesion, and binding of endothelial cells are mediated via STAT3.

## 4. Discussion

In this study, we performed siRNA-mediated gene silencing and RNA sequencing in order to study the autocrine functions of IL6 in cultured human endothelial cells. In contrast to the general assumption that the IL6R*α* is only expressed on leucocytes and hepatocytes [9], we recently showed that human endothelial cells do express functional IL6R*α* on their surface, and that exposure to IL6 causes intracellular signaling in human endothelial cells [5]. We further reported that IL6 classic signaling and transsignaling in human endothelial cells induce distinct molecular signaling events resulting in different cellular responses [5]. In the current study, knockdown of IL6 was performed in the presence and absence of sIL6R*α* in order to study the autocrine functions of IL6 in human endothelial cells under conditions which allow for both classic signaling and transsignaling.

Data obtained in the present study confirm that IL6 acts in an autocrine manner in human endothelial cells, as knockdown of IL6 results in alteration in gene expression of a large number of genes. Furthermore, exposure of endothelial cells to the sIL6R*α* induces an increase in the release of IL6, suggesting that the activation of IL6 transsignaling induces a positive feedback loop, resulting in further synthesis and release of IL6. This concept has been proposed for IL6-mediated transsignaling [26] but has so far not been shown for IL6-mediated classic signaling. We also report here that the mRNA expression of IL6R*α* as well as the signal-transducer gp130 are upregulated after IL6 knockdown. This is in line with what we previously showed on protein level [5] and suggests that the cells compensate the loss of signal from IL6 by increasing the expression of the receptors.

It is well established that the JAK/STAT pathway is an essential signal transduction cascade for many different cytokines [27]. Specifically, JAK1, JAK2, and STAT3 are known to be downstream signaling molecules induced by IL6 [28]. Although their activities are mainly regulated by protein phosphorylations, we report here that they are also transcriptionally regulated in response to IL6 knockdown. SOCS3 is a negative regulator of IL6 signaling [29] and is known to be induced by IL6 signaling [30]. In our data, SOCS3 is downregulated in response to IL6 knockdown, which is not surprising as the IL6 signaling is reduced when the levels of IL6 are decreased.

A large body of evidence has shown that IL6-mediated transsignaling gives rise to a number of proinflammatory responses in different cells [12]. As human endothelial cells produce and release IL6, exposure to sIL6R*α* should in theory activate IL6-mediated transsignaling, and thereby result in altered gene expression of many different genes. However, in this study, only 59 genes were differentially regulated when the cells were treated with recombinant sIL6R*α*. This is in contrast to nearly 2000 genes that were differentially regulated after IL6 knockdown. Furthermore, knockdown of IL6 in the presence and absence of sIL6R*α* resulted in very similar transcriptional patterns suggesting that regulation of most of these genes are independent of the sIL6R*α* receptor. This indicates that the autocrine functions of IL6 in human endothelial cells are mainly mediated via IL6 classic signaling. Our data further suggest that IL6 classic signaling is constantly active in cultured human endothelial cells, as knockdown of IL6 dramatically changes the transcriptional pattern. For three of the top regulated genes after IL6 knockdown (NEK7, TMEM87B, and ESCO2), we also showed that specific blockage of IL6 transsignaling using sgp130Fc [24] does not alter the gene expression of those genes, suggesting that they are indeed regulated by IL6 classic signaling. Although exposure to sIL6R*α* alone resulted in differential expression of 59 genes, it is likely that higher levels of IL6 than the endothelial cells are able to produce are required to induce transsignaling-mediated proinflammatory responses in vascular endothelial cells.

By comparing the differentially regulated genes after IL6 knockdown to public databases (STRING and IPA), we noted that approximately seventy percent of the differentially regulated genes have not been previously reported as first or second neighbors to IL6. When the same comparison was made for the 10% most up- and downregulated genes, a slightly higher proportion of the genes were found among the previously reported first and second neighbors of IL6. However, approximately two-thirds of most differentially expressed genes cannot be found among the first or second neighbors to IL6 in public databases. Hence, in this study, we have identified a large number of genes that previously have not been reported as IL6 regulated. While most previous studies have evaluated the impact of high levels of IL6, mimicking inflammatory conditions, we choose a different approach, where we examined the consequence of reducing the IL6 production from the cells. This approach allowed us to identify novel genes that are under autocrine regulation by IL6 in human endothelial cells.

By using IPA, we found that pathways and functions related to basal cellular functions (such as regulation of cell cycle, signaling, or cellular movement) are regulated as a consequence of IL6 knockdown. Pathways and functions related to cell cycle regulation were inhibited as a response to IL6 knockdown, suggesting that IL6 acts in an autocrine manner to control cell cycle progression. This is in line with previous findings showing that exposure to IL6 promotes cell cycle progression in human endothelial cells [31], as well as in other cell types [32–34]. Furthermore, IL6 knockdown resulted in the activation of functions related to migration and cellular movement, indicating that IL6 acts in an autocrine manner to control cellular movement and migration.

In order to evaluate whether the enriched canonical pathways overlap, we plotted the pathways together with their corresponding genes. As expected, most of the genes in the inhibited pathways were downregulated, while most of the genes in the activated pathways were upregulated. The overlap between the canonical pathways were relatively small, and approximately two-thirds of the genes were unique for one pathway, showing that knockdown of IL6 in human endothelial cells affects several distinct canonical pathways related to signaling and cell cycle regulation.

As we used human vascular endothelial cells as a model to study the autocrine functions of IL6, we further evaluated the enriched functions related to “Cardiovascular system development and function.” All enriched functions in this category were related to binding, adhesion, or interaction of endothelial cells, and all those functions were predicted to be activated upon IL6 knockdown. By combining the differentially regulated genes from these functions with the genes known to be central in IL6-mediated signaling, a merged network was generated. This network shows that the interplay between IL6-mediated signaling and interaction, binding, and adhesion of endothelial cells is mainly mediated via STAT3. Taken together, our data suggest that IL6 acts in an autocrine manner to inhibit binding, adhesion, and interaction of vascular endothelial cells. While high levels of exogenous IL6 have been reported to increase lymphocyte adhesion to endothelial cells [35], our data indicate that the autocrine functions of IL6 may be the opposite and might instead promote a nonadhesive endothelial phenotype.

## 5. Conclusion

This study shows that knockdown of IL6 in human endothelial cells results in dramatic changes in transcriptional pattern, and a large number of novel genes that are under autocrine regulation by IL6 in human endothelial cells were identified. The present study also reveals that the autocrine functions of IL6 in human endothelial cells seem to be related to basal cellular functions such as regulation of cell cycle, signaling, or cellular movement, and suggests that the autocrine functions of IL6 in human endothelial cells are mediated via IL6 classic signaling.

## Figures and Tables

**Table 1:** Knockdown of IL6 in human endothelial cells affect biofunctions related to cardiovascular system development and function. Using Ingenuity Pathway Analysis (IPA), biofunctions regulated by IL6 knockdown in human endothelial cells were evaluated. Activated functions are indicated in orange. Functions with Benjamini-Hochberg's estimated false discovery rate (FDR) < 0.05 and *z* − score<−2 or >2 were considered as enriched.

IPA biofunctions annotation	*z*-score	*p* value	FDR	No. of molecules
Interaction of vascular endothelial cells	**3.130**	2.45*E*‐08	2.07*E*‐06	32
Binding of vascular endothelial cells	**2.993**	7.26*E*‐08	4.89*E*‐06	31
Adhesion of vascular endothelial cells	**2.552**	9.50*E*‐06	2.87*E*‐04	23
Binding of endothelial cells	**2.414**	3.83*E*‐08	2.93*E*‐06	40
Interaction of endothelial cells	**2.404**	6.53*E*‐09	6.29*E*‐07	42

## Data Availability

The datasets generated and analyzed during the current study are available in the NCBI's Gene Expression Omnibus repository [36] and are accessible through GEO Series accession number GSE141783 (https://www.ncbi.nlm.nih.gov/geo/query/acc.cgi?acc=GSE141783).

## Abbreviations

FDR: False discovery rate
HUVECs: Human umbilical vein endothelial cells
IL6: Interleukin 6
IL6R*α*: Interleukin 6 receptor alpha
IL6ST: Interleukin 6 signal transducer
IPA: Ingenuity Pathway Analysis
sgp130Fc: Soluble glycoprotein 130 Fc fusion protein
siRNA: Small interfering RNA
sIL6R*α*: Soluble interleukin 6 receptor alpha
VEGF: Vascular endothelial growth factor.

## Supplementary Materials

Supplementary Materials Supplementary Table 1: top 25 up- and downregulated genes induced by IL6 knockdown in human endothelial cells. Supplementary Table 2: top 25 up- and downregulated genes induced by IL6 knockdown in the presence of soluble IL6 receptor *α* in human endothelial cells. Supplementary Table 3: differentially regulated genes induced by soluble IL6 receptor *α* in human endothelial cells. Supplementary Table 4: knockdown of IL6 in human endothelial cells results in changes in gene expression of proteins known to be involved in IL6-mediated signaling. Supplementary Table 5: canonical pathways enriched as a consequence of IL6 knockdown in human endothelial cells. Supplementary Table 6: diseases and biofunctions enriched as a consequence of IL6 knockdown in human endothelial cells. Supplementary Table 7: novel IL6 regulated genes in human endothelial cells.
