# Supplementary material for: Global Transcriptional Profiling Reveals Novel Autocrine Functions of Interleukin 6 in Human Vascular Endothelial Cells
Source: Mediators Inflamm. 2020 Apr 28;2020:4623107. doi: 10.1155/2020/4623107 (PMC7204122; doi:10.1155/2020/4623107)
Supplement: Supplementary Materials — Supplementary Table 1: top 25 up- and downregulated genes induced by IL6 knockdown in human endothelial cells. Supplementary Table 2: top 25 up- and downregulated genes induced by IL6 knockdown in the presence of soluble IL6 receptor α in human endothelial cells. Supplementary Table 3: differentially regulated genes induced by soluble IL6 receptor α in human endothelial cells. Supplementary Table 4: knockdown of IL6 in human endothelial cells results in changes in gene expression of proteins known to be involved in IL6-mediated signaling. Supplementary Table 5: canonical pathways enriched as a consequence of IL6 knockdown in human endothelial cells. Supplementary Table 6: diseases and biofunctions enriched as a consequence of IL6 knockdown in human endothelial cells. Supplementary Table 7: novel IL6 regulated genes in human endothelial cells. [file 4623107.f1.docx]

Supplementary Table 1 – Top 25 Up- and down regulated genes induced by IL6 knockdown in human endothelial cells.

Supplementary Table 2 – Top 25 Up- and down regulated genes induced by IL6 knockdown in presence of soluble IL6-receptor α in human endothelial cells

Supplementary Table 3 – Differentially regulated genes induced by soluble IL6-receptor α in human endothelial cells

Supplementary Table 4 – Knockdown of IL6 in human endothelial cells results in changes in gene expression of proteins known to be involved in IL6 mediated signaling

Supplementary Table 5 – Canonical pathways enriched as a consequence of IL6 knockdown in human endothelial cells

Supplementary Table 6 - Diseases and Biofunctions enriched as a consequence of IL6 knockdown in human endothelial cells

Supplementary Table 7 - Novel IL6 regulated genes in human endothelial cells.

**Supplementary Table 1 – Top 25 Up and down regulated genes induced by IL6 knockdown**

| **Symbol** | **Entrez Gene Name** | **Log^2^ fold change** | **Fold Change** | **p-value** | **FDR** |
| --- | --- | --- | --- | --- | --- |
| FER1L6 | fer-1 like family member 6 | **3.99** | **15.83** | 1.6E-73 | 2.2E-71 |
| CHRNA1 | cholinergic receptor nicotinic alpha 1 subunit | **3.26** | **9.57** | 2.3E-56 | 1.5E-54 |
| TMEM87B | transmembrane protein 87B | **2.72** | **6.58** | 0.0E+00 | 0.0E+00 |
| NEK7 | NIMA related kinase 7 | **2.64** | **6.21** | 2.4E-285 | 1.6E-281 |
| IL1A | interleukin 1 alpha | **2.50** | **5.65** | 1.4E-79 | 2.5E-77 |
| NEGR1 | neuronal growth regulator 1 | **2.45** | **5.45** | 2.5E-76 | 3.8E-74 |
| LBH | limb bud and heart development | **2.29** | **4.87** | 7.2E-84 | 1.4E-81 |
| GCH1 | GTP cyclohydrolase 1 | **2.27** | **4.83** | 2.2E-94 | 6.4E-92 |
| P4HA3 | prolyl 4-hydroxylase subunit alpha 3 | **2.24** | **4.73** | 2.3E-140 | 2.0E-137 |
| TMEM236 | transmembrane protein 236 | **2.22** | **4.67** | 1.2E-32 | 2.5E-31 |
| ANKRD46 | ankyrin repeat domain 46 | **2.18** | **4.53** | 4.3E-149 | 4.3E-146 |
| KCNN2 | potassium calcium-activated channel subfamily N member 2 | **2.11** | **4.30** | 3.3E-42 | 1.2E-40 |
| DACT1 | dishevelled binding antagonist of beta catenin 1 | **2.09** | **4.25** | 9.9E-38 | 2.8E-36 |
| TGFBR1 | transforming growth factor beta receptor 1 | **2.07** | **4.21** | 9.8E-169 | 1.3E-165 |
| ZNF365 | zinc finger protein 365 | **2.05** | **4.13** | 1.3E-26 | 2.1E-25 |
| TGFB2 | transforming growth factor beta 2 | **1.94** | **3.84** | 4.3E-28 | 7.6E-27 |
| PDZD2 | PDZ domain containing 2 | **1.93** | **3.80** | 1.5E-44 | 6.0E-43 |
| PI16 | peptidase inhibitor 16 | **1.90** | **3.72** | 4.6E-29 | 8.6E-28 |
| RAB3A | RAB3A, member RAS oncogene family | **1.88** | **3.67** | 1.5E-20 | 1.6E-19 |
| ADGRA2 | adhesion G protein-coupled receptor A2 | **1.84** | **3.58** | 3.2E-74 | 4.4E-72 |
| MEDAG | mesenteric estrogen dependent adipogenesis | **1.81** | **3.49** | 5.0E-44 | 1.9E-42 |
| PLEKHA1 | pleckstrin homology domain containing A1 | **1.75** | **3.36** | 2.5E-159 | 2.7E-156 |
| NLRC3 | NLR family CARD domain containing 3 | **1.75** | **3.35** | 1.7E-21 | 2.0E-20 |
| ST5 | suppression of tumorigenicity 5 | **1.74** | **3.34** | 3.3E-68 | 3.5E-66 |
| LGALS3BP | galectin 3 binding protein | **1.74** | **3.34** | 6.6E-18 | 6.2E-17 |
| RINL | Ras and Rab interactor like | **-1.93** | **0.26** | 3.2E-72 | 4.1E-70 |
| FSTL5 | follistatin like 5 | **-1.94** | **0.26** | 6.4E-25 | 9.4E-24 |
| GSDMC | gasdermin C | **-1.98** | **0.25** | 1.6E-28 | 2.9E-27 |
| IDI1 | isopentenyl-diphosphate delta isomerase 1 | **-1.98** | **0.25** | 5.3E-189 | 1.2E-185 |
| MCM10 | minichromosome maintenance 10 replication initiation factor | **-2.01** | **0.25** | 6.0E-94 | 1.7E-91 |
| NUDCD2 | NudC domain containing 2 | **-2.03** | **0.24** | 3.2E-116 | 1.6E-113 |
| SMIM10 | small integral membrane protein 10 | **-2.05** | **0.24** | 3.3E-32 | 7.1E-31 |
| DEPDC1B | DEP domain containing 1B | **-2.10** | **0.23** | 5.3E-104 | 1.9E-101 |
| SLC44A4 | solute carrier family 44 member 4 | **-2.10** | **0.23** | 6.6E-28 | 1.1E-26 |
| CABP4 | calcium binding protein 4 | **-2.11** | **0.23** | 1.2E-49 | 6.0E-48 |
| STMN3 | stathmin 3 | **-2.13** | **0.23** | 4.5E-46 | 1.9E-44 |
| PRRX1 | paired related homeobox 1 | **-2.14** | **0.23** | 1.6E-58 | 1.1E-56 |
| ESCO2 | establishment of sister chromatid cohesion N-acetyltransferase 2 | **-2.20** | **0.22** | 2.0E-65 | 1.9E-63 |
| FAM111B | family with sequence similarity 111 member B | **-2.23** | **0.21** | 1.5E-88 | 3.4E-86 |
| CLEC4GP1 | C-type lectin domain family 4 member G pseudogene 1 | **-2.24** | **0.21** | 8.5E-51 | 4.3E-49 |
| E2F2 | E2F transcription factor 2 | **-2.25** | **0.21** | 9.7E-43 | 3.5E-41 |
| PTPN7 | protein tyrosine phosphatase, non-receptor type 7 | **-2.31** | **0.20** | 2.6E-35 | 6.5E-34 |
| SKP2 | S-phase kinase associated protein 2 | **-2.38** | **0.19** | 7.4E-204 | 3.2E-200 |
| FST | follistatin | **-2.69** | **0.16** | 4.3E-93 | 1.2E-90 |
| CXCL6 | C-X-C motif chemokine ligand 6 | **-2.70** | **0.15** | 8.4E-38 | 2.4E-36 |
| CORIN | corin, serine peptidase | **-2.84** | **0.14** | 5.0E-73 | 6.6E-71 |
| RAB27B | RAB27B, member RAS oncogene family | **-2.90** | **0.13** | 4.9E-56 | 3.2E-54 |
| ABCC3 | ATP binding cassette subfamily C member 3 | **-3.01** | **0.12** | 4.8E-92 | 1.3E-89 |
| ACKR4 | atypical chemokine receptor 4 | **-3.24** | **0.11** | 1.7E-74 | 2.5E-72 |
| SLC7A14 | solute carrier family 7 member 14 | **-3.39** | **0.10** | 4.4E-64 | 3.9E-62 |

FDR: Benjamini-Hochberg false discovery rate

**Supplementary Table 2 – Top 25 Up- and down regulated genes induced by IL6 knockdown in presence of soluble IL6-receptor α**

| **Symbol** | **Entrez Gene Name** | **Log^2^ Fold change** | **Fold Change** | **p-value** | **FDR** |
| --- | --- | --- | --- | --- | --- |
| FER1L6 | fer-1 like family member 6 | **4.02** | **16.17** | 7.2E-66 | 3.3E-64 |
| CHRNA1 | cholinergic receptor nicotinic alpha 1 subunit | **3.65** | **12.51** | 6.1E-88 | 5.2E-86 |
| NEK7 | NIMA related kinase 7 | **2.85** | **7.21** | 0.0E+00 | 0.0E+00 |
| NEGR1 | neuronal growth regulator 1 | **2.83** | **7.09** | 6.8E-175 | 4.0E-172 |
| TMEM87B | transmembrane protein 87B | **2.75** | **6.74** | 0.0E+00 | 0.0E+00 |
| IL1A | interleukin 1 alpha | **2.70** | **6.50** | 1.3E-109 | 1.9E-107 |
| TMEM236 | transmembrane protein 236 | **2.58** | **5.97** | 6.0E-36 | 9.7E-35 |
| LBH | limb bud and heart development | **2.40** | **5.29** | 4.1E-158 | 1.6E-155 |
| P4HA3 | prolyl 4-hydroxylase subunit alpha 3 | **2.37** | **5.16** | 0.0E+00 | 0.0E+00 |
| APOL4 | apolipoprotein L4 | **2.35** | **5.09** | 5.4E-45 | 1.2E-43 |
| TGFBR1 | transforming growth factor beta receptor 1 | **2.26** | **4.80** | 3.7E-158 | 1.5E-155 |
| ANKRD46 | ankyrin repeat domain 46 | **2.21** | **4.62** | 1.1E-213 | 1.1E-210 |
| ZNF365 | zinc finger protein 365 | **2.19** | **4.57** | 2.6E-39 | 4.9E-38 |
| GCH1 | GTP cyclohydrolase 1 | **2.19** | **4.57** | 4.2E-164 | 1.8E-161 |
| KCNN2 | potassium calcium-activated channel subfamily N member 2 | **2.19** | **4.56** | 1.6E-46 | 3.9E-45 |
| MEDAG | mesenteric estrogen dependent adipogenesis | **2.17** | **4.51** | 5.8E-142 | 1.6E-139 |
| TGFB2 | transforming growth factor beta 2 | **2.13** | **4.36** | 1.5E-27 | 1.7E-26 |
| NLRC3 | NLR family CARD domain containing 3 | **2.06** | **4.17** | 1.8E-30 | 2.3E-29 |
| PLEKHA1 | pleckstrin homology domain containing A1 | **1.85** | **3.61** | 8.4E-234 | 1.1E-230 |
| COL12A1 | collagen type XII alpha 1 chain | **1.85** | **3.59** | 1.1E-117 | 1.9E-115 |
| CASP12 | caspase 12 (gene/pseudogene) | **1.84** | **3.59** | 4.4E-34 | 6.5E-33 |
| GUCY1A2 | guanylate cyclase 1 soluble subunit alpha 2 | **1.80** | **3.48** | 8.1E-79 | 5.4E-77 |
| ASXL3 | additional sex combs like 3, transcriptional regulator | **1.79** | **3.45** | 2.1E-60 | 8.0E-59 |
| DUSP8 | dual specificity phosphatase 8 | **1.77** | **3.42** | 2.1E-43 | 4.6E-42 |
| CHSY3 | chondroitin sulfate synthase 3 | **1.77** | **3.41** | 7.9E-90 | 7.2E-88 |
| ADIRF-AS1 | ADIRF antisense RNA 1 | **-1.93** | **0.26** | 9.1E-55 | 3.0E-53 |
| FAM111B | family with sequence similarity 111 member B | **-1.95** | **0.26** | 4.1E-135 | 1.0E-132 |
| SELL | selectin L | **-1.99** | **0.25** | 2.1E-33 | 3.0E-32 |
| IDI1 | isopentenyl-diphosphate delta isomerase 1 | **-1.99** | **0.25** | 2.7E-246 | 4.3E-243 |
| EGR1 | early growth response 1 | **-2.02** | **0.25** | 2.7E-38 | 4.9E-37 |
| NUDCD2 | NudC domain containing 2 | **-2.03** | **0.25** | 1.5E-154 | 5.2E-152 |
| SLCO4A1 | solute carrier organic anion transporter family member 4A1 | **-2.06** | **0.24** | 8.7E-58 | 3.1E-56 |
| NAALADL1 | N-acetylated alpha-linked acidic dipeptidase like 1 | **-2.08** | **0.24** | 2.0E-46 | 4.8E-45 |
| TXNIP | thioredoxin interacting protein | **-2.08** | **0.24** | 2.0E-93 | 2.1E-91 |
| SMIM10 | small integral membrane protein 10 | **-2.15** | **0.23** | 5.9E-60 | 2.3E-58 |
| KIF19 | kinesin family member 19 | **-2.16** | **0.22** | 7.1E-43 | 1.5E-41 |
| FST | follistatin | **-2.19** | **0.22** | 8.6E-52 | 2.6E-50 |
| E2F2 | E2F transcription factor 2 | **-2.20** | **0.22** | 3.4E-60 | 1.3E-58 |
| RINL | Ras and Rab interactor like | **-2.20** | **0.22** | 7.2E-119 | 1.3E-116 |
| PTPN7 | protein tyrosine phosphatase, non-receptor type 7 | **-2.30** | **0.20** | 1.1E-47 | 2.9E-46 |
| CABP4 | calcium binding protein 4 | **-2.34** | **0.20** | 8.0E-54 | 2.5E-52 |
| SKP2 | S-phase kinase associated protein 2 | **-2.35** | **0.20** | 2.4E-255 | 5.3E-252 |
| CLEC4GP1 | C-type lectin domain family 4 member G pseudogene 1 | **-2.54** | **0.17** | 8.2E-68 | 4.1E-66 |
| RAB27B | RAB27B, member RAS oncogene family | **-2.61** | **0.16** | 4.2E-55 | 1.4E-53 |
| CXCL6 | C-X-C motif chemokine ligand 6 | **-2.68** | **0.16** | 2.7E-73 | 1.5E-71 |
| CORIN | corin, serine peptidase | **-2.73** | **0.15** | 3.2E-106 | 4.4E-104 |
| STMN3 | stathmin 3 | **-2.83** | **0.14** | 1.4E-102 | 1.8E-100 |
| ABCC3 | ATP binding cassette subfamily C member 3 | **-2.93** | **0.13** | 7.3E-114 | 1.2E-111 |
| ACKR4 | atypical chemokine receptor 4 | **-2.96** | **0.13** | 1.2E-65 | 5.5E-64 |
| SLC7A14 | solute carrier family 7 member 14 | **-3.11** | **0.12** | 1.9E-41 | 3.9E-40 |

FDR: Benjamini-Hochberg false discovery rate

**Supplementary Table 3 – All 59 differentially regulated genes induced by soluble IL6-receptor α**

| **Symbol** | **Entrez Gene Name** | **Log^2^ fold change** | **Fold change** | **p-value** | **BH q-value** |
| --- | --- | --- | --- | --- | --- |
| HGF | hepatocyte growth factor | **1.59** | **3.00** | 7.9E-18 | 1.7E-14 |
| CCL23 | C-C motif chemokine ligand 23 | **1.40** | **2.64** | 2.8E-22 | 8.9E-19 |
| SELE | selectin E | **1.37** | **2.59** | 6.6E-09 | 1.7E-06 |
| CORIN | corin. serine peptidase | **1.37** | **2.58** | 1.3E-22 | 5.7E-19 |
| SOCS3 | suppressor of cytokine signaling 3 | **1.35** | **2.54** | 1.6E-53 | 2.0E-49 |
| TNR | tenascin R | **1.31** | **2.49** | 1.0E-16 | 1.7E-13 |
| TNIP3 | TNFAIP3 interacting protein 3 | **1.31** | **2.47** | 3.2E-09 | 8.5E-07 |
| JAK3 | Janus kinase 3 | **1.28** | **2.43** | 1.5E-27 | 1.0E-23 |
| SLCO2A1 | solute carrier organic anion transporter family member 2A1 | **1.16** | **2.23** | 5.6E-14 | 6.1E-11 |
| SELP | selectin P | **1.15** | **2.22** | 4.9E-19 | 1.3E-15 |
| CXCL2 | C-X-C motif chemokine ligand 2 | **1.08** | **2.11** | 2.4E-12 | 1.6E-09 |
| CEBPD | CCAAT/enhancer binding protein delta | **1.06** | **2.08** | 8.2E-14 | 7.6E-11 |
| ACP5 | acid phosphatase 5. tartrate resistant | **1.04** | **2.05** | 1.2E-15 | 1.7E-12 |
| RAB3C | RAB3C. member RAS oncogene family | **0.98** | **1.97** | 2.2E-15 | 2.9E-12 |
| SELL | selectin L | **0.93** | **1.91** | 3.4E-11 | 1.3E-08 |
| FAM167B | family with sequence similarity 167 member B | **0.91** | **1.87** | 3.7E-10 | 1.2E-07 |
| IL1R1 | interleukin 1 receptor type 1 | **0.87** | **1.83** | 4.8E-12 | 2.6E-09 |
| HSD17B2 | hydroxysteroid 17-beta dehydrogenase 2 | **0.87** | **1.82** | 2.4E-11 | 9.5E-09 |
| C1R | complement C1r | **0.86** | **1.82** | 5.5E-08 | 1.2E-05 |
| SULF1 | sulfatase 1 | **0.83** | **1.78** | 5.4E-07 | 8.6E-05 |
| FAP | fibroblast activation protein alpha | **0.82** | **1.76** | 2.3E-10 | 7.7E-08 |
| IL33 | interleukin 33 | **0.80** | **1.74** | 2.6E-09 | 7.0E-07 |
| IFITM1 | interferon induced transmembrane protein 1 | **0.79** | **1.73** | 1.3E-08 | 3.1E-06 |
| POSTN | periostin | **0.79** | **1.72** | 1.3E-11 | 6.0E-09 |
| CLIC2 | chloride intracellular channel 2 | **0.78** | **1.71** | 8.2E-06 | 1.0E-03 |
| IL6 | interleukin 6 | **0.76** | **1.70** | 2.0E-13 | 1.6E-10 |
| CCL14 | C-C motif chemokine ligand 14 | **0.76** | **1.69** | 3.9E-07 | 6.9E-05 |
| RHOU | ras homolog family member U | **0.76** | **1.69** | 1.6E-08 | 3.7E-06 |
| CCL2 | C-C motif chemokine ligand 2 | **0.73** | **1.66** | 6.5E-14 | 6.5E-11 |
| CFI | complement factor I | **0.73** | **1.65** | 5.6E-14 | 6.1E-11 |
| GLT8D2 | glycosyltransferase 8 domain containing 2 | **0.71** | **1.64** | 2.1E-06 | 2.9E-04 |
| TNFSF10 | tumor necrosis factor superfamily member 10 | **0.71** | **1.63** | 7.4E-10 | 2.2E-07 |
| KIF19 | kinesin family member 19 | **0.69** | **1.62** | 8.8E-07 | 1.3E-04 |
| ZNF385D | zinc finger protein 385D | **0.69** | **1.61** | 8.7E-06 | 1.0E-03 |
| IGFBP1 | insulin like growth factor binding protein 1 | **0.68** | **1.60** | 8.7E-05 | 7.2E-03 |
| TTC39A | tetratricopeptide repeat domain 39A | **0.68** | **1.60** | 6.0E-08 | 1.3E-05 |
| SEMA7A | semaphorin 7A (John Milton Hagen blood group) | **0.67** | **1.59** | 5.6E-05 | 5.0E-03 |
| CXCL3 | C-X-C motif chemokine ligand 3 | **0.66** | **1.58** | 2.5E-05 | 2.6E-03 |
| ANKRD55 | ankyrin repeat domain 55 | **0.65** | **1.57** | 1.6E-09 | 4.7E-07 |
| ANKRD29 | ankyrin repeat domain 29 | **0.64** | **1.56** | 8.7E-07 | 1.3E-04 |
| MGP | matrix Gla protein | **0.64** | **1.55** | 1.1E-16 | 1.7E-13 |
| ALDH1A1 | aldehyde dehydrogenase 1 family member A1 | **0.63** | **1.55** | 1.0E-12 | 7.7E-10 |
| LYRM1 | LYR motif containing 1 | **0.63** | **1.55** | 3.6E-12 | 2.2E-09 |
| LINC01091 | long intergenic non-protein coding RNA 1091 | **0.63** | **1.55** | 1.9E-05 | 2.1E-03 |
| OSMR | oncostatin M receptor | **0.62** | **1.54** | 1.9E-11 | 8.0E-09 |
| IFI44L | interferon induced protein 44 like | **0.62** | **1.54** | 4.9E-04 | 3.1E-02 |
| PCDH7 | protocadherin 7 | **0.62** | **1.54** | 1.3E-12 | 9.2E-10 |
| AQP3 | aquaporin 3 (Gill blood group) | **0.61** | **1.53** | 1.2E-05 | 1.4E-03 |
| CMAHP | cytidine monophospho-N-acetylneuraminic acid hydroxylase, pseudogene | **0.60** | **1.52** | 6.9E-06 | 8.7E-04 |
| FLRT3 | fibronectin leucine rich transmembrane protein 3 | **0.59** | **1.51** | 2.5E-07 | 4.7E-05 |
| MX1 | MX dynamin like GTPase 1 | **0.59** | **1.50** | 5.2E-04 | 3.2E-02 |
| TRPC4 | transient receptor potential cation channel subfamily C member 4 | **-0.60** | **0.66** | 5.8E-05 | 5.1E-03 |
| WNT9A | Wnt family member 9A | **-0.62** | **0.65** | 1.4E-05 | 1.6E-03 |
| APCDD1L | APC down-regulated 1 like | **-0.65** | **0.64** | 4.4E-07 | 7.6E-05 |
| EPHA5 | EPH receptor A5 | **-0.67** | **0.63** | 9.7E-05 | 7.9E-03 |
| MYEOV | myeloma overexpressed | **-0.71** | **0.61** | 5.6E-12 | 2.9E-09 |
| SLC30A3 | solute carrier family 30 member 3 | **-0.73** | **0.60** | 3.2E-06 | 4.3E-04 |
| MMP10 | matrix metallopeptidase 10 | **-0.74** | **0.60** | 3.9E-12 | 2.2E-09 |
| DNAH8 | dynein axonemal heavy chain 8 | **-0.77** | **0.59** | 2.4E-08 | 5.5E-06 |

FDR: Benjamini-Hochberg false discovery rate

| **Supplementary Table 4 – Knockdown of IL6 in human endothelial cells results in changes in gene expression of proteins known to be involved in IL6 mediated signaling** | | | | | |
| --- | --- | --- | --- | --- | --- |
| **Gene** | **Entrez Gene Name** | **Log^2^ Fold Change** | **Fold Change** | **p-value** | **FDR** |
| IL6 | interleukin L6 | **-1.23** | **0.43** | 6.0E-21 | 6.8E-20 |
| IL6R | interleukin 6 receptor | **0.22** | **1.17** | 1.2E-03 | 2.4E-03 |
| IL6ST | interleukin 6 signal transducer/glycoprotein 130 | **1.01** | **2.01** | 1.3E-46 | 5.5E-45 |
| STAT3 | signal transducer and activator of transcription 3 | **0.25** | **1.19** | 2.7E-06 | 8.2E-06 |
| JAK1 | janus kinase 1 | **0.35** | **1.28** | 1.6E-09 | 7.3E-09 |
| JAK2 | janus kinase 2 | **0.20** | **1.15** | 3.2E-03 | 6.1E-03 |
| SOCS3 | suppressor of cytokine signaling 3 | **-0.80** | **0.58** | 2.8E-29 | 5.2E-28 |

FDR: Benjamini-Hochberg false discovery rate.

| **Supplementary Table 5 – Enriched canonical pathways due to IL6 knockdown in human endothelial cells** | | | | | | | | | | | |
| --- | --- | --- | --- | --- | --- | --- | --- | --- | --- | --- | --- |
|  | **CTL vs IL6KD** | | | | | **sIL6R vs IL6 KD + sIL6R** | | | | | **Total number of genes in pathway** |
|  |  | **Regulated genes** | |  |  |  | **Regulated genes** | |  |  |  |
| **Canonical Pathway** | **z-score** | **number** | **%** | **p-value** | **FDR** | **z-score** | **number** | **%** | **p-value** | **FDR** |  |
| Mitotic Roles of Polo-Like Kinase | **-3.13** | 24 | 38 | **3.81E-10** | **9.52E-8** | **-2.83** | 22 | 35 | **1.71E-8** | **4.29E-6** | 63 |
| PTEN Signaling | **-2.52** | 19 | 16 | **1.05E-2** | **n.s.** | **-2.67** | 17 | 14 | **4.40E-2** | **n.s.** | 119 |
| Role of BRCA1 in DNA Damage Response | **-2.50** | 23 | 29 | **2.27E-7** | **2.27E-5** | **-2.32** | 21 | 27 | **4.71E-6** | **4.70E-4** | 78 |
| Cyclins and Cell Cycle Regulation | **-2.32** | 18 | 23 | **1.40E-4** | **5.37E-3** | **-2.67** | 17 | 22 | **5.07E-4** | **1.45E-2** | 77 |
| PPAR Signaling | **-1.39** | 13 | 14 | **6.16E-2** | **n.s.** | **-2.14** | 14 | 16 | **3.44E-2** | **n.s.** | 90 |
| Glioma Signaling | **1.61** | 24 | 22 | **3.36E-5** | **n.s.** | **2.06** | 23 | 21 | **1.19E-4** | **5.38E-3** | 109 |
| Protein Kinase A Signaling | **1.64** | 46 | 12 | **2.22E-2** | **n.s.** | **2.41** | 46 | 12 | **2.70E-2** | **n.s.** | 376 |
| IL-8 Signaling | **2.04** | 24 | 12 | **n.s.** | **n.s.** | **2.35** | 26 | 13 | **3.58E-2** | **n.s.** | 196 |
| Cell Cycle: G2/M DNA Damage Checkpoint Regulation | **2.18** | 18 | 37 | **1.15E-7** | **1.44E-5** | **2.00** | 17 | 35 | **8.00E-7** | **1.33E-4** | 49 |
| PI3K Signaling in B Lymphocytes | **2.18** | 21 | 17 | **3.70E-3** | **n.s.** | **2.14** | 18 | 15 | **3.40E-2** | **n.s.** | 124 |
| Leukocyte Extravasation Signaling | **2.29** | 26 | 13 | **4.98E-2** | **n.s.** | **1.63** | 27 | 13 | **3.56E-2** | **n.s.** | 205 |
| Glioblastoma Multiforme Signaling | **2.29** | 24 | 22 | **9.50E-4** | **1.98E-2** | **2.45** | 28 | 26 | **5.23E-5** | **1.45E-2** | 109 |
| Integrin Signaling | **2.45** | 27 | 13 | **4.44E-2** | **n.s.** | **2.45** | 27 | 13 | **5.07E-2** | **n.s.** | 212 |
| Glioma Invasiveness Signaling | **2.71** | 11 | 16 | **4.92E-2** | **2.71E-2** | **2.32** | 15 | 21 | **1.46E-3** | **2.71E-2** | 70 |
| Cardiac Hypertrophy Signaling | **3.40** | 28 | 12 | **n.s.** | **n.s.** | **2.47** | 32 | 14 | **1.30E-2** | **n.s.** | 232 |
| FDR: Benjamini-Hochberg false discovery rate. N/A: not available. n.s: not significant. Genes with a fold change 1.5 (log fold change 0.584962500721156) and FDR of 0.05 are included in the core analysis. Canonical pathways with a p-value <0.05 and z-score: <-2 or > 2 after IL6 Knock-down either in the absence or presence of sIL6R are considered as enriched. Blue values indicate inhibited pathways. and orange values indicate activated pathways. | | | | | | | | | | | |
|  | | | | | | | | | | | |
|  | | | | | | | | | | | |

| \| **Supplementary Table 6 - Enriched diseases and Biofunctions due to IL6 knockdown in human endothelial cells** \| \| \| \| \| \| \| \| \| \| \| --- \| --- \| --- \| --- \| --- \| --- \| --- \| --- \| --- \| --- \| \|  \|  \| **CTL vs IL6 KD** \| \| \| \| **sIL6R vs IL6 KD + sIL6R** \| \| \| \| \|  \|  \| **z-score** \| **p** \| **FDR** \| **No of molecules** \| **z-score** \| **p** \| **FDR** \| **No of molecules** \| \| **Cell cycle** \| \|  \|  \|  \|  \|  \|  \|  \|  \| \|  \| cytokinesis \| **-3.542** \| 3.30E-10 \| 4.21E-08 \| 40 \| **-3.344** \| 6.22E-09 \| 6.62E-07 \| 38 \| \|  \| M phase of tumor cell lines \| **-3.488** \| 1.76E-12 \| 3.92E-10 \| 36 \| **-3.36** \| 2.86E-10 \| 3.89E-08 \| 33 \| \|  \| M phase \| **-3.439** \| 1.76E-17 \| 4.72E-14 \| 60 \| **-3.209** \| 2.56E-15 \| 3.15E-12 \| 57 \| \|  \| interphase \| **-2.965** \| 3.50E-13 \| 9.62E-11 \| 118 \| **-3.013** \| 8.48E-13 \| 2.89E-10 \| 118 \| \|  \| G2/M phase \| **-2.781** \| 6.59E-08 \| 4.46E-06 \| 42 \| **-3.118** \| 2.67E-07 \| 1.55E-05 \| 41 \| \|  \| cytokinesis of tumor cell lines \| **-2.762** \| 2.41E-07 \| 1.34E-05 \| 21 \| **-2.584** \| 2.29E-05 \| 7.01E-04 \| 18 \| \|  \| G2 phase \| **-2.733** \| 4.01E-09 \| 4.17E-07 \| 53 \| **-2.898** \| 1.71E-08 \| 1.55E-06 \| 52 \| \|  \| interphase of tumor cell lines \| **-2.651** \| 6.02E-10 \| 7.32E-08 \| 86 \| **-2.596** \| 5.60E-09 \| 6.02E-07 \| 84 \| \|  \| cycling of centrosome \| **-2.424** \| 1.27E-11 \| 2.47E-09 \| 26 \| **-2.787** \| 1.15E-10 \| 1.66E-08 \| 25 \| \|  \| M phase of cervical cancer cell lines \| **-2.409** \| 3.82E-08 \| 2.93E-06 \| 24 \| **-2.198** \| 3.77E-06 \| 1.64E-04 \| 21 \| \|  \| cytokinesis of cervical cancer cell lines \| **-2.236** \| 1.44E-05 \| 3.98E-04 \| 16 \| **N/A** \| n.s. \| n.s. \| - \| \|  \| S phase \| **-2.064** \| 9.59E-15 \| 5.13E-12 \| 56 \| **-1.525** \| 6.55E-14 \| 4.18E-11 \| 55 \| \|  \| segregation of chromosomes \| **-1.806** \| 3.26E-20 \| 1.16E-16 \| 45 \| **-2.09** \| 4.10E-19 \| 1.40E-15 \| 44 \| \|  \| formation of mitotic spindle \| **N/A** \| n.s \| n.s \| - \| **2.138** \| 3.60E-05 \| 1.01E-03 \| 17 \| \|  \| senescence of cells \| **2.631** \| 1.57E-06 \| 6.80E-05 \| 45 \| **3.003** \| 2.74E-05 \| 8.08E-04 \| 42 \| \|  \|  \|  \|  \|  \|  \|  \|  \|  \|  \| \| **Cellular movement** \| \|  \|  \|  \|  \|  \|  \|  \|  \| \|  \| cytokinesis \| **-3.542** \| 3.30E-10 \| 4.21E-08 \| 40 \| **-3.344** \| 6.22E-09 \| 6.62E-07 \| 38 \| \|  \| cytokinesis of tumor cell lines \| **-2.762** \| 2.41E-07 \| 1.34E-05 \| 21 \| **-2.584** \| 2.29E-05 \| 7.01E-04 \| 18 \| \|  \| cytokinesis of cervical cancer cell lines \| **-2.236** \| 1.44E-05 \| 3.98E-04 \| 16 \| **N/A** \| n.s \| n.s \| - \| \|  \| cell movement of brain cancer cell lines \| **1.337** \| 4.93E-09 \| 4.95E-07 \| 39 \| **2.025** \| 6.95E-07 \| 3.55E-05 \| 35 \| \|  \| migration of tumor cells \| **1.448** \| 5.83E-08 \| 4.06E-06 \| 23 \| **2.883** \| 1.17E-08 \| 1.11E-06 \| 147 \| \|  \| migration of brain cancer cell lines \| **1.609** \| 6.42E-08 \| 4.38E-06 \| 33 \| **2.490** \| 2.38E-05 \| 7.26E-04 \| 28 \| \|  \| invasion of cells \| **1.750** \| 5.64E-14 \| 2.16E-11 \| 163 \| **2.225** \| 8.44E-14 \| 4.38E-11 \| 164 \| \|  \| migration of connective tissue cells \| **1.812** \| 5.24E-09 \| 5.20E-07 \| 19 \| **2.136** \| 1.62E-06 \| 7.73E-05 \| 16 \| \|  \| cell movement of connective tissue cells \| **1.871** \| 1.63E-09 \| 1.82E-07 \| 23 \| **2.189** \| 3.38E-07 \| 1.93E-05 \| 20 \| \|  \| invasion of tumor cell lines \| **1.946** \| 6.32E-13 \| 1.57E-10 \| 149 \| **2.570** \| 1.75E-12 \| 4.71E-10 \| 149 \| \|  \| migration of cells \| **2.021** \| 3.44E-11 \| 6.14E-09 \| 224 \| **2.642** \| 2.41E-11 \| 4.40E-09 \| 227 \| \|  \| cell movement of blood cells \| **2.126** \| 4.39E-07 \| 2.21E-05 \| 69 \| **N/A** \| n.s \| n.s \| - \| \|  \| leukocyte migration \| **2.229** \| 4.84E-07 \| 2.42E-05 \| 68 \| **1.700** \| 1.25E-05 \| 4.41E-04 \| 64 \| \|  \| cell movement of tumor cell lines \| **2.272** \| 5.08E-12 \| 1.05E-09 \| 181 \| **2.828** \| 2.85E-11 \| 5.02E-09 \| 180 \| \|  \| cell movement \| **2.284** \| 3.88E-12 \| 8.31E-10 \| 253 \| **2.827** \| 3.68E-13 \| 1.30E-10 \| 260 \| \|  \| cell movement of fibroblasts \| **2.431** \| 2.91E-07 \| 1.57E-05 \| 15 \| **2.431** \| 2.28E-06 \| 1.04E-04 \| 14 \| \|  \|  \|  \|  \|  \|  \|  \|  \|  \|  \| \| **DNA Replication. Recombination. and Repair** \| \|  \|  \|  \|  \|  \|  \|  \|  \| \|  \| alignment of chromosomes \| **-3.195** \| 8.60E-17 \| 1.32E-13 \| 19 \| **-3.195** \| 1.10E-16 \| 2.80E-13 \| 19 \| \|  \| repair of DNA \| **-3.122** \| 4.99E-08 \| 3.63E-06 \| 57 \| **-3.663** \| 4.99E-06 \| 2.01E-04 \| 52 \| \|  \| DNA replication \| **-2.591** \| 3.18E-09 \| 3.37E-07 \| 42 \| **-2.390** \| 1.42E-09 \| 1.63E-07 \| 43 \| \|  \| chromosomal congression of chromosomes \| **-2.538** \| 3.59E-11 \| 6.30E-09 \| 11 \| **-2.538** \| 4.14E-11 \| 6.82E-09 \| 11 \| \|  \| metabolism of DNA \| **-2.422** \| 2.38E-08 \| 2.05E-06 \| 57 \| **-2.439** \| 5.24E-07 \| 2.82E-05 \| 54 \| \|  \| segregation of chromosomes \| **-1.806** \| 3.26E-20 \| 1.16E-16 \| 45 \| **-2.090** \| 4.10E-19 \| 1.40E-15 \| 44 \| \|  \| formation of nuclear foci \| **2.141** \| 1.22E-06 \| 5.43E-05 \| 22 \| **N/A** \| n.s \| n.s \| - \| \|  \| quantity of mitotic spindle \| **2.425** \| 1.60E-05 \| 4.29E-04 \| 8 \| **N/A** \| n.s \| n.s \| - \| \|  \| formation of gamma H2AX nuclear focus \| **2.902** \| 3.50E-06 \| 1.32E-04 \| 18 \| **N/A** \| n.s \| n.s \| - \| \|  \|  \|  \|  \|  \|  \|  \|  \|  \|  \| \| **Cellular Assembly and Organization** \| \|  \|  \|  \|  \|  \|  \|  \|  \| \|  \| alignment of chromosomes \| **-3.195** \| 8.60E-17 \| 1.32E-13 \| 19 \| **-3.195** \| 1.10E-16 \| 2.80E-13 \| 19 \| \|  \| association of chromatin \| **-2.773** \| 1.60E-05 \| 4.29E-04 \| 8 \| **N/A** \| n.s \| n.s \| - \| \|  \| chromosomal congression of chromosomes \| **-2.538** \| 3.59E-11 \| 6.30E-09 \| 11 \| **-2.538** \| 4.14E-11 \| 6.82E-09 \| 11 \| \|  \| formation of nuclear foci \| **2.141** \| 1.22E-06 \| 5.43E-05 \| 22 \| **N/A** \| n.s \| n.s \| - \| \|  \| quantity of mitotic spindle \| **2.425** \| 1.60E-05 \| 4.29E-04 \| 8 \| **N/A** \| n.s \| n.s \| - \| \|  \| formation of gamma H2AX nuclear focus \| **2.902** \| 3.50E-06 \| 1.32E-04 \| 18 \| **N/A** \| n.s \| n.s \| - \| \|  \|  \|  \|  \|  \|  \|  \|  \|  \|  \| \| **Cell-To-Cell Signaling and Interaction** \| \|  \|  \|  \|  \|  \|  \|  \|  \| \|  \| interaction of endothelial cells \| **2.404** \| 6.53E-09 \| 6.29E-07 \| 42 \| **1.445** \| 8.92E-08 \| 6.28E-06 \| 40 \| \|  \| binding of endothelial cells \| **2.414** \| 3.83E-08 \| 2.93E-06 \| 40 \| **1.435** \| 4.70E-07 \| 2.55E-05 \| 38 \| \|  \| adhesion of vascular endothelial cells \| **2.552** \| 9.50E-06 \| 2.87E-04 \| 23 \| **1.626** \| 3.46E-06 \| 1.52E-04 \| 24 \| \|  \| binding of vascular endothelial cells \| **2.993** \| 7.26E-08 \| 4.89E-06 \| 31 \| **2.301** \| 9.75E-08 \| 6.77E-06 \| 31 \| \|  \| interaction of vascular endothelial cells \| **3.130** \| 2.45E-08 \| 2.07E-06 \| 32 \| **2.457** \| 3.33E-08 \| 2.73E-06 \| 32 \| \|  \|  \|  \|  \|  \|  \|  \|  \|  \|  \| \| **Cardiovascular System Development and Function** \| \|  \|  \|  \|  \|  \|  \|  \|  \| \|  \| interaction of endothelial cells \| **2.404** \| 6.53E-09 \| 6.29E-07 \| 42 \| **1.445** \| 8.92E-08 \| 6.28E-06 \| 40 \| \|  \| binding of endothelial cells \| **2.414** \| 3.83E-08 \| 2.93E-06 \| 40 \| **1.435** \| 4.70E-07 \| 2.55E-05 \| 38 \| \|  \| adhesion of vascular endothelial cells \| **2.552** \| 9.50E-06 \| 2.87E-04 \| 23 \| **1.626** \| 3.46E-06 \| 1.52E-04 \| 24 \| \|  \| binding of vascular endothelial cells \| **2.993** \| 7.26E-08 \| 4.89E-06 \| 31 \| **2.301** \| 9.75E-08 \| 6.77E-06 \| 31 \| \|  \| interaction of vascular endothelial cells \| **3.130** \| 2.45E-08 \| 2.07E-06 \| 32 \| **2.457** \| 3.33E-08 \| 2.73E-06 \| 32 \| \|  \|  \|  \|  \|  \|  \|  \|  \|  \|  \| \| **Cell Morphology** \| \|  \|  \|  \|  \|  \|  \|  \|  \| \|  \| formation of mitotic spindle \| **N/A** \| n.s \| n.s \| - \| **2.138** \| 3.60E-05 \| 1.01E-03 \| 17 \| \|  \| cell spreading \| **1.769** \| 1.59E-07 \| 9.24E-06 \| 39 \| **2.03** \| 7.16E-05 \| 1.74E-03 \| 33 \| \|  \|  \|  \|  \|  \|  \|  \|  \|  \|  \| \| **Cellular development** \| \|  \|  \|  \|  \|  \|  \|  \|  \| \|  \| cell proliferation of carcinoma cell lines \| **-2.092** \| 8.27E-08 \| 5.50E-06 \| 83 \| **-1.69** \| 3.46E-08 \| 2.80E-06 \| 85 \| \|  \|  \|  \|  \|  \|  \|  \|  \|  \|  \| \| **Cellular Growth and Proliferation** \| \|  \|  \|  \|  \|  \|  \|  \|  \| \|  \| cell proliferation of carcinoma cell lines \| **-2.092** \| 8.27E-08 \| 5.50E-06 \| 83 \| **-1.69** \| 3.46E-08 \| 2.80E-06 \| 85 \| \|  \|  \|  \|  \|  \|  \|  \|  \|  \|  \| \| **Connective Tissue Development and Function** \| \|  \|  \|  \|  \|  \|  \|  \|  \| \|  \| cell movement of fibroblasts \| **2.431** \| 2.91E-07 \| 1.57E-05 \| 15 \| **2.431** \| 2.28E-06 \| 1.04E-04 \| 14 \| \|  \|  \|  \|  \|  \|  \|  \|  \|  \|  \| \| **Immune Cell Trafficking** \| \|  \|  \|  \|  \|  \|  \|  \|  \| \|  \| leukocyte migration \| **2.229** \| 4.84E-07 \| 2.42E-05 \| 68 \| **1.700** \| 1.25E-05 \| 4.41E-04 \| 64 \| \|  \|  \|  \|  \|  \|  \|  \|  \|  \|  \| \| FDR: Benjamini-Hochberg false discovery rate. N/A: not available. n.s: not significant. Genes with a fold change 1.5 (log fold change 0.584962500721156) and Benjamini Hochberg FDR of 0.05 were included in the core analysis. Diseases and biofunctions with a Benjamini Hochberg FDR <0.05 and z-score: <-2 or > 2 after IL6 Knock-down either in the absence or presence of sIL6R are considered as enriched. Blue values indicate inhibited functions and orange values indicate activated functions. \| \| \| \| \| \| \| \| \| \| \|  \| \| \| \| \| \| \| \| \| \| \|  \| \| \| \| \| \| \| \| \| \| |
| --- | --- | --- | --- | --- | --- | --- | --- | --- | --- | --- | --- | --- | --- | --- | --- | --- | --- | --- | --- | --- | --- | --- | --- | --- | --- | --- | --- | --- | --- | --- | --- | --- | --- | --- | --- | --- | --- | --- | --- | --- | --- | --- | --- | --- | --- | --- | --- | --- | --- | --- | --- | --- | --- | --- | --- | --- | --- | --- | --- | --- | --- | --- | --- | --- | --- | --- | --- | --- | --- | --- | --- | --- | --- | --- | --- | --- | --- | --- | --- | --- | --- | --- | --- | --- | --- | --- | --- | --- | --- | --- | --- | --- | --- | --- | --- | --- | --- | --- | --- | --- | --- | --- | --- | --- | --- | --- | --- | --- | --- | --- | --- | --- | --- | --- | --- | --- | --- | --- | --- | --- | --- | --- | --- | --- | --- | --- | --- | --- | --- | --- | --- | --- | --- | --- | --- | --- | --- | --- | --- | --- | --- | --- | --- | --- | --- | --- | --- | --- | --- | --- | --- | --- | --- | --- | --- | --- | --- | --- | --- | --- | --- | --- | --- | --- | --- | --- | --- | --- | --- | --- | --- | --- | --- | --- | --- | --- | --- | --- | --- | --- | --- | --- | --- | --- | --- | --- | --- | --- | --- | --- | --- | --- | --- | --- | --- | --- | --- | --- | --- | --- | --- | --- | --- | --- | --- | --- | --- | --- | --- | --- | --- | --- | --- | --- | --- | --- | --- | --- | --- | --- | --- | --- | --- | --- | --- | --- | --- | --- | --- | --- | --- | --- | --- | --- | --- | --- | --- | --- | --- | --- | --- | --- | --- | --- | --- | --- | --- | --- | --- | --- | --- | --- | --- | --- | --- | --- | --- | --- | --- | --- | --- | --- | --- | --- | --- | --- | --- | --- | --- | --- | --- | --- | --- | --- | --- | --- | --- | --- | --- | --- | --- | --- | --- | --- | --- | --- | --- | --- | --- | --- | --- | --- | --- | --- | --- | --- | --- | --- | --- | --- | --- | --- | --- | --- | --- | --- | --- | --- | --- | --- | --- | --- | --- | --- | --- | --- | --- | --- | --- | --- | --- | --- | --- | --- | --- | --- | --- | --- | --- | --- | --- | --- | --- | --- | --- | --- | --- | --- | --- | --- | --- | --- | --- | --- | --- | --- | --- | --- | --- | --- | --- | --- | --- | --- | --- | --- | --- | --- | --- | --- | --- | --- | --- | --- | --- | --- | --- | --- | --- | --- | --- | --- | --- | --- | --- | --- | --- | --- | --- | --- | --- | --- | --- | --- | --- | --- | --- | --- | --- | --- | --- | --- | --- | --- | --- | --- | --- | --- | --- | --- | --- | --- | --- | --- | --- | --- | --- | --- | --- | --- | --- | --- | --- | --- | --- | --- | --- | --- | --- | --- | --- | --- | --- | --- | --- | --- | --- | --- | --- | --- | --- | --- | --- | --- | --- | --- | --- | --- | --- | --- | --- | --- | --- | --- | --- | --- | --- | --- | --- | --- | --- | --- | --- | --- | --- | --- | --- | --- | --- | --- | --- | --- | --- | --- | --- | --- | --- | --- | --- | --- | --- | --- | --- | --- | --- | --- | --- | --- | --- | --- | --- | --- | --- | --- | --- | --- | --- | --- | --- | --- | --- | --- | --- | --- | --- | --- | --- | --- | --- | --- | --- | --- | --- | --- | --- | --- | --- | --- | --- | --- | --- | --- | --- | --- | --- | --- | --- | --- | --- | --- | --- | --- | --- | --- | --- | --- | --- | --- | --- | --- | --- | --- | --- | --- | --- | --- | --- | --- | --- | --- | --- | --- | --- | --- | --- | --- | --- | --- | --- | --- | --- | --- | --- | --- | --- | --- | --- | --- | --- | --- | --- | --- | --- | --- | --- | --- | --- | --- | --- | --- | --- | --- | --- | --- | --- | --- | --- | --- | --- | --- | --- | --- | --- | --- | --- | --- | --- | --- | --- | --- | --- | --- | --- | --- | --- | --- | --- | --- | --- | --- | --- | --- | --- | --- | --- | --- | --- | --- | --- | --- | --- | --- | --- | --- | --- | --- | --- | --- | --- | --- | --- | --- | --- | --- | --- | --- | --- | --- | --- | --- | --- | --- | --- | --- | --- | --- | --- | --- | --- | --- | --- | --- | --- | --- | --- | --- | --- | --- | --- | --- | --- | --- | --- | --- | --- | --- | --- | --- | --- | --- | --- | --- | --- | --- | --- | --- | --- | --- | --- | --- | --- | --- | --- | --- | --- | --- | --- | --- | --- | --- | --- | --- | --- | --- | --- | --- | --- | --- | --- | --- | --- | --- | --- | --- | --- | --- | --- | --- | --- | --- | --- | --- | --- | --- | --- | --- | --- | --- | --- | --- | --- | --- | --- | --- | --- | --- | --- | --- | --- | --- | --- | --- | --- | --- | --- | --- | --- | --- | --- | --- | --- | --- | --- | --- | --- | --- | --- | --- | --- | --- | --- | --- | --- | --- | --- | --- | --- | --- | --- | --- | --- | --- | --- | --- | --- | --- | --- | --- | --- | --- | --- | --- | --- | --- | --- | --- | --- | --- | --- | --- | --- | --- | --- | --- | --- | --- | --- | --- | --- | --- | --- | --- | --- | --- | --- | --- | --- | --- | --- | --- | --- | --- | --- | --- | --- | --- | --- | --- | --- | --- | --- | --- | --- | --- | --- | --- | --- | --- | --- | --- | --- | --- | --- | --- | --- | --- | --- | --- | --- | --- | --- | --- | --- | --- | --- | --- | --- | --- | --- | --- | --- | --- | --- | --- | --- | --- | --- | --- | --- | --- | --- | --- | --- | --- | --- | --- | --- | --- | --- | --- | --- | --- | --- | --- | --- | --- | --- | --- | --- | --- | --- | --- | --- | --- | --- | --- | --- | --- | --- | --- | --- | --- | --- | --- | --- | --- | --- | --- | --- | --- | --- | --- | --- | --- | --- | --- | --- | --- | --- | --- | --- | --- | --- | --- | --- | --- | --- | --- | --- | --- |

**Supplementary Table 7 - Novel IL6 regulated genes in human endothelial cells**

Genes differentially regulated after IL6 knockdown in human endothelial cell, but not described as interaction partners in IPA or STRING. Red indicates upregulated genes, green indicates down-regulated genes.

| **AATBC** | **ANO2** | **BRMS1L** | **CCDC163** | **CHAMP1** | **DCLRE1A** | **ESM1** | **GABPA** |
| --- | --- | --- | --- | --- | --- | --- | --- |
| **ABAT** | **ANO4** | **BTN2A1** | **CCDC18** | **CHST1** | **DCLRE1B** | **ETFDH** | **GABRR2** |
| **ABCA2** | **ANO7** | **C11orf95** | **CCDC183-AS1** | **CHST11** | **DDAH1** | **EVA1A** | **GAL** |
| **ABCA4** | **ANO8** | **C12orf49** | **CCDC188** | **CHST14** | **DDIAS** | **EXO1** | **GALNS** |
| **ABCA7** | **ANTXR1** | **C12orf65** | **CCDC18-AS1** | **CHSY1** | **DDR2** | **EYA3** | **GALNT12** |
| **ABCC3** | **AOC2** | **C12orf75** | **CCDC190** | **CHSY3** | **DDX12P** | **F2RL1** | **GALNT15** |
| **ABHD13** | **AOX1** | **C12orf76** | **CCDC191** | **CKAP2L** | **DDX24** | **FAM102B** | **GAPLINC** |
| **ABHD14B** | **AP1G2** | **C14orf119** | **CCDC34** | **CLDN12** | **DENND6B** | **FAM109A** | **GAS6** |
| **ABHD17C** | **AP4S1** | **C14orf37** | **CCDC36** | **CLDN14** | **DERA** | **FAM111A** | **GATA6-AS1** |
| **ABHD3** | **APBA1** | **C15orf54** | **CCDC57** | **CLEC4GP1** | **DERL3** | **FAM111B** | **GBE1** |
| **ABHD5** | **APCDD1** | **C16orf45** | **CCDC58** | **CLIC4** | **DGCR2** | **FAM120AOS** | **GBP4** |
| **ABHD8** | **APLNR** | **C16orf58** | **CCDC68** | **CLIP1** | **DGKD** | **FAM120C** | **GCKR** |
| **ABI3BP** | **APLP1** | **C17orf53** | **CCDC74A** | **CLMP** | **DHRS11** | **FAM124A** | **GCOM1** |
| **ACAP3** | **APOBEC3B** | **C17orf75** | **CCDC77** | **CLPB** | **DHX35** | **FAM126B** | **GDAP1** |
| **ACBD7** | **AREL1** | **C18orf54** | **CCDC80** | **CLSPN** | **DKK3** | **FAM129A** | **GDF3** |
| **ACE** | **ARFGEF3** | **C19orf48** | **CCDC88B** | **CLSTN1** | **DNA2** | **FAM13A-AS1** | **GEMIN2** |
| **ACKR4** | **ARHGAP21** | **C1orf112** | **CCL23** | **CLSTN3** | **DNAH8** | **FAM160B1** | **GEN1** |
| **ACLY** | **ARHGAP45** | **C1orf226** | **CCL28** | **CMAHP** | **DNAJB4** | **FAM167B** | **GFOD1** |
| **ACRBP** | **ARHGAP5-AS1** | **C1RL** | **CCM2L** | **CNOT6** | **DNAJC12** | **FAM171A2** | **GGACT** |
| **ACSM3** | **ARHGEF17** | **C1RL-AS1** | **CCNB3** | **CNPY4** | **DNAJC22** | **FAM185A** | **GGPS1** |
| **ADAM12** | **ARHGEF39** | **C21orf2** | **CCNF** | **COL12A1** | **DNAJC3-AS1** | **FAM208A** | **GIMAP4** |
| **ADAM22** | **ARID5A** | **C21orf58** | **CCNL2** | **COL25A1** | **DNAJC4** | **FAM212A** | **GINS1** |
| **ADAM32** | **ARL13B** | **C22orf39** | **CD109** | **COL4A2** | **DNM3** | **FAM216A** | **GINS2** |
| **ADAM9** | **ARL5A** | **C2CD5** | **CD2AP** | **COL6A1** | **DNMBP-AS1** | **FAM219A** | **GINS3** |
| **ADAMTS10** | **ARMC4** | **C2orf27** | **CD302** | **COL6A2** | **DNPH1** | **FAM221A** | **GINS4** |
| **ADAMTS12** | **ARMT1** | **C3orf58** | **CD34** | **COLEC12** | **DOK3** | **FAM229A** | **GIPC3** |
| **ADAMTS18** | **ARRDC1-AS1** | **C3orf70** | **CD9** | **COLGALT1** | **DSCC1** | **FAM234B** | **GK** |
| **ADAMTS3** | **ARRDC4** | **C4orf46** | **CD99** | **COQ6** | **DSEL** | **FAM26E** | **GLCCI1** |
| **ADAMTS4** | **ARSB** | **C4orf48** | **CDC42EP5** | **CORIN** | **DUSP18** | **FAM3C2** | **GLIDR** |
| **ADAMTS7** | **ASCC3** | **C5orf24** | **CDC42SE2** | **COX15** | **E2F5** | **FAM46A** | **GLIS3** |
| **ADAMTSL4** | **ASF1B** | **C5orf30** | **CDCA3** | **COX7A1** | **E2F8** | **FAM64A** | **GLO1** |
| **ADCY4** | **ASXL3** | **C5orf34** | **CDCA7** | **CPA4** | **ECE1** | **FAM69A** | **GNG11** |
| **ADGRA2** | **ATAD5** | **C5orf63** | **CDH12** | **CPEB2** | **ECHDC2** | **FAM72A** | **GNPDA1** |
| **ADGRF5** | **ATG4D** | **C8orf37** | **CDH13** | **CPNE5** | **ECT2** | **FAM72B** | **GNS** |
| **ADIRF** | **ATL1** | **C8orf4** | **CDK5RAP2** | **CPNE7** | **EDA** | **FAM83D** | **GOLIM4** |
| **ADIRF-AS1** | **ATP11A** | **C9orf116** | **CDKAL1** | **CREB3L1** | **EDIL3** | **FAM86HP** | **GOLPH3L** |
| **ADPRH** | **ATP13A2** | **C9orf172** | **CDKL5** | **CREB5** | **EFCAB11** | **FBXL19-AS1** | **GOLT1B** |
| **ADRA1D** | **ATP13A3** | **CA8** | **CDKN2AIPNL** | **CREG1** | **EFEMP2** | **FBXO21** | **GPALPP1** |
| **ADSSL1** | **ATP6AP1** | **CABP4** | **CENPH** | **CRISPLD1** | **EFR3A** | **FBXO36** | **GPAT2** |
| **AGO4** | **ATP6AP2** | **CACNA2D1** | **CENPI** | **CRLF3** | **EGFL7** | **FER1L4** | **GPAT3** |
| **AGPAT4** | **ATP6V0E2** | **CACNG6** | **CENPJ** | **CRTAC1** | **EGFLAM** | **FER1L6** | **GPR137C** |
| **AIFM2** | **ATP6V1G1** | **CACUL1** | **CENPO** | **CSGALNACT1** | **EHD3** | **FERMT1** | **GPR146** |
| **AKIRIN1** | **ATP7B** | **CADM3** | **CENPP** | **CST1** | **EIF4E2** | **FGF16** | **GPR153** |
| **ALCAM** | **AUNIP** | **CALM1** | **CENPV** | **CTC-338M12.4** | **EIF4E3** | **FIRRE** | **GPR155** |
| **ALDH6A1** | **AVIL** | **CAMK1** | **CENPW** | **CTDP1** | **ELOVL4** | **FKBP9** | **GPR160** |
| **ALG10** | **AVL9** | **CAMSAP2** | **CEP120** | **CTDSP2** | **EMC3-AS1** | **FLRT2** | **GPR176** |
| **ALG9** | **B3GNT5** | **CAND2** | **CEP128** | **CTHRC1** | **EMCN** | **FNIP1** | **GPR3** |
| **ALPK1** | **B3GNT7** | **CAPS** | **CEP135** | **CTSZ** | **EMILIN2** | **FOLH1** | **GPR4** |
| **AMD1** | **B3GNT9** | **CARNMT1** | **CEP152** | **CTTNBP2NL** | **EML4** | **FOXD2-AS1** | **GPRIN3** |
| **AMIGO1** | **B4GALT4** | **CASC15** | **CEP19** | **CTXN1** | **ENDOD1** | **FOXL1** | **GPSM1** |
| **AMMECR1** | **BAIAP2L1** | **CASC4** | **CEP295** | **CWC22** | **ENTPD4** | **FOXN2** | **GPSM3** |
| **AMPH** | **BBX** | **CASK** | **CEP57** | **CXADR** | **ENTPD7** | **FOXP2** | **GRIK5** |
| **ANGPTL2** | **BCCIP** | **CASP12** | **CEP63** | **CYB561** | **EOGT** | **FRRS1** | **GRPEL2** |
| **ANK3** | **BCHE** | **CBR4** | **CEP83** | **CYB5R4** | **EPB41L1** | **FSTL3** | **GSDMB** |
| **ANKFY1** | **BEST3** | **CCBE1** | **CERCAM** | **CYP2S1** | **EPHB6** | **FSTL5** | **GSDMC** |
| **ANKH** | **BEX5** | **CCDC125** | **CERK** | **CYP2U1** | **EPSTI1** | **FUNDC1** | **GSG2** |
| **ANKRD18A** | **BLVRB** | **CCDC136** | **CFAP44** | **CYTL1** | **ERCC6L** | **FUNDC2** | **GSTCD** |
| **ANKRD18B** | **BMP2K** | **CCDC138** | **CFAP45** | **CYTOR** | **ERI1** | **FURIN** | **GTF2F2** |
| **ANKRD36C** | **BMPR1B** | **CCDC14** | **CFAP46** | **DACT1** | **ERLIN1** | **FUT8** | **GTF2H3** |
| **ANKRD46** | **BNC2** | **CCDC149** | **CGREF1** | **DAW1** | **ERMP1** | **FYTTD1** | **GUCY1A2** |
| **ANKRD52** | **BPNT1** | **CCDC150** | **CHAC2** | **DCHS1** | **ERV3-1** | **FZD2** | **GUCY1B3** |
| **ANKRD55** | **BRI3BP** | **CCDC152** | **CHAF1B** | **DCK** | **ESCO2** | **GABBR2** | **GYPC** |

| **GZF1** | **KCNN2** | **LINC01776** | **LZTS3** | **MTBP** | **NUP35** | **PIGN** | **PRR11** |
| --- | --- | --- | --- | --- | --- | --- | --- |
| **H1F0** | **KCNN3** | **LITAF** | **MAFB** | **MTFR1** | **NXPE3** | **PIGS** | **PRR5** |
| **H1FX-AS1** | **KCTD20** | **LMAN2L** | **MAGI2-AS3** | **MTFR2** | **NXPH3** | **PIP4K2C** | **PRRG1** |
| **HAGLROS** | **KCTD3** | **LMBR1L** | **MALSU1** | **MTMR9** | **OCIAD2** | **PITHD1** | **PRRT3** |
| **HDAC11** | **KDELC1** | **LMF1** | **MAMLD1** | **MTPN** | **ODC1** | **PITPNC1** | **PRRX1** |
| **HDGFRP3** | **KHDC1** | **LMLN** | **MAN1A1** | **MTRF1** | **OGFRL1** | **PITPNM1** | **PRTFDC1** |
| **HES2** | **KHK** | **LMO7** | **MAN1B1** | **MTUS1** | **OIP5** | **PITPNM3** | **PSMB8-AS1** |
| **HFE** | **KHNYN** | **LMTK2** | **MAN2A1** | **MTX3** | **OLFML3** | **PKD1L1** | **PSMC3IP** |
| **HIC1** | **KIAA0586** | **LNX1** | **MAN2B2** | **MYEOV** | **OLMALINC** | **PLAC9** | **PSMG3-AS1** |
| **HIST2H2BE** | **KIAA1211L** | **LOC100049716** | **MANEA** | **MYO15B** | **OPRL1** | **PLAG1** | **PSPH** |
| **HIVEP2** | **KIAA1324L** | **LOC100379224** | **MANEAL** | **MYO1E** | **ORMDL3** | **PLAT** | **PSRC1** |
| **HJURP** | **KIAA1614** | **LOC100499484** | **MANSC1** | **MYO7A** | **OSBPL6** | **PLCB1** | **PTCD3** |
| **HLA-DPA1** | **KIF13A** | **LOC100505501** | **MAP1A** | **MYPN** | **OSCP1** | **PLCB2** | **PTER** |
| **HLA-DPB1** | **KIF14** | **LOC100505549** | **MAP1S** | **MYRIP** | **OTUD6B-AS1** | **PLCD1** | **PTGR2** |
| **HMCN1** | **KIF18B** | **LOC100505771** | **MAP3K21** | **NAALADL1** | **P3H2** | **PLCD4** | **PTK7** |
| **HMG20A** | **KIF19** | **LOC100506127** | **MAPK6** | **NAB1** | **P4HA3** | **PLCL1** | **PTMAP5** |
| **HMGN2P5** | **KIF1A** | **LOC100506302** | **MAPK8IP1** | **NAF1** | **PAFAH2** | **PLEKHA1** | **PTP4A2P2** |
| **HMGXB4** | **KIF20B** | **LOC100506473** | **MARK4** | **NALCN** | **PAIP1** | **PLEKHA3** | **PTPN18** |
| **HNMT** | **KIF26B** | **LOC100506691** | **MARVELD2** | **NAP1L5** | **PALD1** | **PLEKHA4** | **PTPN21** |
| **HNRNPA1P10** | **KIF3A** | **LOC101927151** | **MAST3** | **NARS2** | **PALM** | **PLEKHA6** | **PTPN7** |
| **HNRNPCP2** | **KIF3B** | **LOC101927204** | **MATN2** | **NBEA** | **PANK3** | **PLEKHA7** | **PTPRE** |
| **HPDL** | **KIF4A** | **LOC101929494** | **MB21D1** | **NCAM2** | **PANX1** | **PLEKHB2** | **PTPRN2** |
| **HS6ST3** | **KIFC1** | **LOC102724434** | **MBNL3** | **NCEH1** | **PAOX** | **PLEKHO1** | **PTPRS** |
| **HSD17B2** | **KLF15** | **LOC103611081** | **MBP** | **NDN** | **PAPLN** | **PLGRKT** | **PURG** |
| **HSD3B7** | **KLF3** | **LOC105369187** | **MC1R** | **NDRG3** | **PAPPA2** | **PLLP** | **PXMP4** |
| **HTR2B** | **KLF8** | **LOC105374952** | **MCRIP1** | **NEBL** | **PAQR3** | **PLPP4** | **PXYLP1** |
| **HTR7P1** | **KLHL2** | **LOC105376805** | **ME3** | **NECTIN2** | **PAQR4** | **PLS1** | **RAB18** |
| **HTRA1** | **KLHL8** | **LOC105376896** | **MED28** | **NEGR1** | **PAQR6** | **PLSCR4** | **RAB27A** |
| **ICA1L** | **KNSTRN** | **LOC105378753** | **MEDAG** | **NEIL3** | **PARD6G** | **PLVAP** | **RAB27B** |
| **IDI1** | **KREMEN1** | **LOC155060** | **MEG3** | **NEK7** | **PARN** | **PLXNA4** | **RAB2B** |
| **IDS** | **KRT80** | **LOC283140** | **MEG9** | **NEMP1** | **PARP14** | **PLXND1** | **RAB30** |
| **IGF2** | **KSR2** | **LOC339803** | **METRN** | **NFIA-AS2** | **PARPBP** | **PM20D2** | **RAB30-AS1** |
| **IGF2BP2** | **KTN1-AS1** | **LOC643072** | **METTL2A** | **NHLRC3** | **PATJ** | **PMM1** | **RAB33A** |
| **IGFBP4** | **KYNU** | **LOC728673** | **MFSD13A** | **NID1** | **PBRM1** | **PNMA2** | **RAB36** |
| **IGSF8** | **LACE1** | **LOXL1** | **MFSD3** | **NID2** | **PCBD1** | **POC1A** | **RAB38** |
| **IKZF2** | **LAMA3** | **LPCAT2** | **MGARP** | **NIN** | **PCDH12** | **POLQ** | **RAB3A** |
| **IL16** | **LAMP3** | **LPP** | **MGAT4B** | **NIPAL1** | **PCDH17** | **POLR1B** | **RAB8B** |
| **IL17RE** | **LAMTOR3** | **LPXN** | **MGST1** | **NKILA** | **PCDH19** | **POLR3GL** | **RABEP1** |
| **IL1RL1** | **LANCL1** | **LRIG3** | **MICAL2** | **NLGN2** | **PCDHGA4** | **POP5** | **RAC1P2** |
| **IL32** | **LANCL3** | **LRP10** | **MICU2** | **NLGN4Y** | **PCDHGB5** | **POU4F1** | **RAD51AP1** |
| **IL33** | **LAYN** | **LRP4** | **MIGA1** | **NLRC3** | **PCGF3** | **PPA1** | **RAD54L** |
| **IMMP2L** | **LBH** | **LRRC3** | **mir-126** | **NLRP14** | **PCGF5** | **PPAT** | **RAET1E** |
| **IMPG2** | **LCAT** | **LRRC34** | **mir-15** | **NLRP2** | **PCK2** | **PPFIBP2** | **RALA** |
| **INCENP** | **LEPROT** | **LRRC71** | **mir-568** | **NMB** | **PCLO** | **PPM1F** | **RAPGEF4** |
| **INPP4A** | **LHFP** | **LRRC8B** | **MIS18BP1** | **NMRAL2P** | **PCNX1** | **PPM1H** | **RARG** |
| **INPP5A** | **LIMCH1** | **LRRCC1** | **MLF1** | **NOL11** | **PCSK5** | **PPM1K** | **RASAL3** |
| **INTS13** | **LIMD2** | **LSM12P1** | **MMP11** | **NOL4L** | **PDE4D** | **PPP1R12A** | **RASGEF1A** |
| **IPMK** | **LIMS2** | **LSM14A** | **MMP15** | **NOP16** | **PDGFD** | **PPP1R16B** | **RASGRF2** |
| **IPO11** | **LIN54** | **LSM3** | **MMP17** | **NORAD** | **PDLIM1** | **PPP1R3F** | **RASGRP3** |
| **IQCA1** | **LIN7B** | **LSM5** | **MNS1** | **NOS1AP** | **PDS5A** | **PPP1R9A** | **RASL11A** |
| **IQCB1** | **LINC00094** | **LSM6** | **MORF4L1P1** | **NPM1P39** | **PECAM1** | **PPP3CB** | **RBM24** |
| **ISPD** | **LINC00106** | **LTB4R** | **MREG** | **NPPA-AS1** | **PECR** | **PPP3CB-AS1** | **RC3H2** |
| **ITGA10** | **LINC00176** | **LTB4R2** | **MRM1** | **NPR2** | **PER3** | **PPWD1** | **RCAN2** |
| **ITGA11** | **LINC00520** | **LTBP2** | **MROH6** | **NPTN** | **PGM2L1** | **PQLC2L** | **RCAN3** |
| **ITGB1P1** | **LINC00565** | **LTBP3** | **MRPL57** | **NPTXR** | **PGM5P2** | **PRCP** | **RCN3** |
| **ITGBL1** | **LINC00639** | **LURAP1** | **MRVI1** | **NRG3** | **PHF14** | **PRDM4** | **RDH10** |
| **ITM2A** | **LINC00899** | **LXN** | **MS4A6A** | **NSMCE4A** | **PHF2** | **PRDM8** | **REC8** |
| **ITPRIPL1** | **LINC00920** | **LYAR** | **MSMP** | **NT5DC2** | **PHF5A** | **PRIM2** | **RECK** |
| **JADE2** | **LINC01106** | **LYSMD1** | **MSR1** | **NTSR1** | **PHKA1** | **PRKAG2** | **REEP3** |
| **JHDM1D-AS1** | **LINC01239** | **LYSMD2** | **MSS51** | **NUDCD1** | **PHTF2** | **PRKCDBP** | **REEP5** |
| **KCNAB2** | **LINC01270** | **LYSMD3** | **MSX1** | **NUF2** | **PI16** | **PROSC** | **REEP6** |
| **KCNC3** | **LINC01341** | **LYVE1** | **MT1E** | **NUP210** | **PIF1** | **PRPF40B** | **RENBP** |

| **RFC2** | **SELENOF** | **SLIT3** | **TAF8** | **TOR1B** | **VWFP1** |
| --- | --- | --- | --- | --- | --- |
| **RFX2** | **SELENOM** | **SMIM10** | **TBC1D31** | **TP53I11** | **XIRP2** |
| **RFX5** | **SEMA3A** | **SMIM13** | **TBXA2R** | **TP53I13** | **XPR1** |
| **RFX7** | **SEMA3G** | **SMIM14** | **TCF19** | **TRAIP** | **XRCC2** |
| **RFXAP** | **SEMA4B** | **SNAPC3** | **TCIRG1** | **TRBC2** | **XRCC3** |
| **RGPD3** | **SEMA7A** | **SNED1** | **TCTE3** | **TRERF1** | **XXYLT1-AS2** |
| **RGS7BP** | **SEPT10** | **SNHG1** | **TDRD9** | **TRIB2** | **YAF2** |
| **RHBDF1** | **SEPT5** | **SNHG10** | **TENM3** | **TRIM16L** | **YIPF1** |
| **RHPN1-AS1** | **SERPIND1** | **SNHG19** | **TES** | **TRIM2** | **YJEFN3** |
| **RINL** | **SESN3** | **SNHG3** | **TESK2** | **TRIM8** | **YKT6** |
| **RIT1** | **SFXN1** | **SNHG8** | **TET1** | **TRIP13** | **YPEL2** |
| **RMI1** | **SFXN3** | **SNORA73B** | **TEX15** | **TRIQK** | **YY2** |
| **RMI2** | **SGO1** | **SNRNP27** | **TFEC** | **TRMT10B** | **ZBTB41** |
| **RNASEH2C** | **SGO2** | **SNRNP40** | **TGFBI** | **TROAP** | **ZBTB46** |
| **RNASET2** | **SH2D4A** | **SNRPG** | **TGFBR3** | **TRPC4** | **ZBTB47** |
| **RNF122** | **SH3RF1** | **SNTA1** | **TICRR** | **TRPM4** | **ZC3H12C** |
| **RNF125** | **SHCBP1** | **SNTB1** | **TIE1** | **TRPM7** | **ZC3HAV1L** |
| **RNF130** | **SHISA2** | **SNX16** | **TIGD2** | **TSPAN11** | **ZCCHC24** |
| **RNF138** | **SHROOM3** | **SNX5** | **TIMELESS** | **TSPAN12** | **ZDHHC1** |
| **RNF144B** | **SIDT2** | **SORT1** | **TIPRL** | **TSPAN14** | **ZFHX2** |
| **RNF152** | **SIRPB2** | **SOWAHC** | **TK2** | **TSPAN15** | **ZGRF1** |
| **RNPEPL1** | **SKA1** | **SOX18** | **TLK2** | **TSPAN6** | **ZKSCAN1** |
| **ROBO3** | **SKA3** | **SPAG5** | **TM6SF1** | **TSPEAR-AS1** | **ZNF117** |
| **ROBO4** | **SLAIN1** | **SPATA20** | **TMA7** | **TSPEAR-AS2** | **ZNF25** |
| **ROGDI** | **SLC12A4** | **SPATA2L** | **TMCC1** | **TSSC1** | **ZNF260** |
| **ROR1** | **SLC15A3** | **SPC24** | **TMCC1-AS1** | **TST** | **ZNF268** |
| **RPARP-AS1** | **SLC15A4** | **SPINK5** | **TMCO4** | **TSTD1** | **ZNF280C** |
| **RPL22L1** | **SLC16A2** | **SPOPL** | **TMED10** | **TTBK2** | **ZNF300** |
| **RPL39L** | **SLC16A6** | **SPRY4** | **TMEM106A** | **TTC32** | **ZNF365** |
| **RPP25L** | **SLC17A5** | **SPX** | **TMEM106C** | **TTC39A** | **ZNF41** |
| **RPSAP52** | **SLC1A1** | **SQRDL** | **TMEM140** | **TTPAL** | **ZNF423** |
| **RRP15** | **SLC22A4** | **SRBD1** | **TMEM147-AS1** | **TUB** | **ZNF454** |
| **RTN2** | **SLC25A12** | **SREK1IP1** | **TMEM154** | **TUBGCP4** | **ZNF467** |
| **RUSC1** | **SLC25A19** | **SRFBP1** | **TMEM156** | **TXLNB** | **ZNF471** |
| **S1PR2** | **SLC25A21** | **SSH2** | **TMEM171** | **TYW5** | **ZNF487** |
| **SALL2** | **SLC25A24** | **ST5** | **TMEM175** | **UBE2D4** | **ZNF491** |
| **SAMD12** | **SLC25A25-AS1** | **ST8SIA4** | **TMEM181** | **UBE2W** | **ZNF503** |
| **SAMD14** | **SLC25A34** | **STARD7-AS1** | **TMEM184B** | **UBL7-AS1** | **ZNF519** |
| **SAP30L** | **SLC27A1** | **STEAP2** | **TMEM2** | **UBP1** | **ZNF589** |
| **SAPCD2** | **SLC27A5** | **STIL** | **TMEM200A** | **UBTD1** | **ZNF620** |
| **SAR1A** | **SLC29A2** | **STK11IP** | **TMEM204** | **UBXN2B** | **ZNF664** |
| **SARM1** | **SLC29A4** | **STRIP2** | **TMEM220** | **UHMK1** | **ZNF677** |
| **SASS6** | **SLC35G1** | **STX11** | **TMEM233** | **UNC93B1** | **ZNF680** |
| **SATB2** | **SLC36A1** | **STYK1** | **TMEM236** | **USB1** | **ZNF699** |
| **SBF2-AS1** | **SLC38A5** | **STYX** | **TMEM245** | **USF3** | **ZNF704** |
| **SCAMP1** | **SLC39A11** | **SUCLG2** | **TMEM255B** | **UTP18** | **ZNF708** |
| **SCAMP1-AS1** | **SLC39A6** | **SUGT1P1** | **TMEM26** | **UTP20** | **ZNF91** |
| **SCARB2** | **SLC40A1** | **SULT1B1** | **TMEM261** | **UTP23** | **ZNF92** |
| **SCARF2** | **SLC41A2** | **SULT1E1** | **TMEM50A** | **WASF1** | **ZSWIM5** |
| **SCIN** | **SLC44A4** | **SUN2** | **TMEM50B** | **VASH2** | **ZXDA** |
| **SCPEP1** | **SLC46A3** | **SUV39H2** | **TMEM63B** | **WDFY4** | **ZYG11A** |
| **SCUBE3** | **SLC47A1** | **SWT1** | **TMEM64** | **WDR24** |  |
| **SDC1** | **SLC6A6** | **SYBU** | **TMEM65** | **WDR54** |  |
| **SDF4** | **SLC7A11-AS1** | **SYNJ2** | **TMEM69** | **WDR62** |  |
| **SDHAF2** | **SLC7A14** | **SYNJ2BP** | **TMEM86B** | **WDR76** |  |
| **SDSL** | **SLC7A8** | **SYNM** | **TMEM87B** | **VGLL3** |  |
| **SEC13** | **SLC9A5** | **SYT1** | **TMEM8A** | **WHRN** |  |
| **SEC22C** | **SLC9A9** | **SYT17** | **TMTC3** | **VPS9D1** |  |
| **SEC24A** | **SLCO2A1** | **TACC3** | **TMX4** | **WRB** |  |
| **SEC63** | **SLCO4A1** | **TADA2B** | **TNRC6C-AS1** | **VRK1** |  |
| **SEL1L3** | **SLFN11** | **TAF10** | **TOMM34** | **VSIG10L** |  |
| **SELENBP1** | **SLFN13** | **TAF7L** | **TOP1MT** | **VSIR** |  |
